# B-Lymphocyte Depletion in Myalgic Encephalopathy/ Chronic Fatigue Syndrome. An Open-Label Phase II Study with Rituximab Maintenance Treatment

**DOI:** 10.1371/journal.pone.0129898

**Published:** 2015-07-01

**Authors:** Øystein Fluge, Kristin Risa, Sigrid Lunde, Kine Alme, Ingrid Gurvin Rekeland, Dipak Sapkota, Einar Kleboe Kristoffersen, Kari Sørland, Ove Bruland, Olav Dahl, Olav Mella

**Affiliations:** 1 Department of Oncology and Medical Physics, Haukeland University Hospital, Bergen, Norway; 2 Department of Clinical Medicine, University of Bergen, Haukeland University Hospital, Bergen, Norway; 3 Department of Immunology and Transfusion Medicine, Haukeland University Hospital, Bergen, Norway; 4 Department of Clinical Science, University of Bergen, Haukeland University Hospital, Bergen, Norway; 5 Department of Medical Genetics and Molecular Medicine, Haukeland University Hospital, Bergen, Norway; Tilburg University, NETHERLANDS

## Abstract

**Background:**

Myalgic Encephalopathy/Chronic Fatigue Syndrome (ME/CFS) is a disease of unknown etiology. We previously reported a pilot case series followed by a small, randomized, placebo-controlled phase II study, suggesting that B-cell depletion using the monoclonal anti-CD20 antibody rituximab can yield clinical benefit in ME/CFS.

**Methods:**

In this single-center, open-label, one-armed phase II study (NCT01156909), 29 patients were included for treatment with rituximab (500 mg/m^2^) two infusions two weeks apart, followed by maintenance rituximab infusions after 3, 6, 10 and 15 months, and with follow-up for 36 months.

**Findings:**

Major or moderate responses, predefined as lasting improvements in self-reported *Fatigue score*, were detected in 18 out of 29 patients (intention to treat). Clinically significant responses were seen in 18 out of 28 patients (64%) receiving rituximab maintenance treatment. For these 18 patients, the mean response durations within the 156 weeks study period were 105 weeks in 14 major responders, and 69 weeks in four moderate responders. At end of follow-up (36 months), 11 out of 18 responding patients were still in ongoing clinical remission. For major responders, the mean lag time from first rituximab infusion until start of clinical response was 23 weeks (range 8–66). Among the nine patients from the placebo group in the previous randomized study with no significant improvement during 12 months follow-up after saline infusions, six achieved a clinical response before 12 months after rituximab maintenance infusions in the present study. Two patients had an allergic reaction to rituximab and two had an episode of uncomplicated late-onset neutropenia. Eight patients experienced one or more transient symptom flares after rituximab infusions. There was no unexpected toxicity.

**Conclusion:**

In a subgroup of ME/CFS patients, prolonged B-cell depletion with rituximab maintenance infusions was associated with sustained clinical responses. The observed patterns of delayed responses and relapse after B-cell depletion and regeneration, a three times higher disease prevalence in women than in men, and a previously demonstrated increase in B-cell lymphoma risk for elderly ME/CFS patients, suggest that ME/CFS may be a variant of an autoimmune disease.

**Trial registration:**

ClinicalTrials.gov NCT01156909

## Introduction

Myalgic Encephalopathy/Chronic Fatigue Syndrome (ME/CFS) is a disease of unknown etiology characterized by severe fatigue and post-exertional malaise, cognitive disturbances, pain, sleep problems, sensory hypersensitivity and several symptoms related to immune and autonomic function. ME/CFS according to Canadian diagnostic criteria [1] comprises approximately 0.1–0.2% of the population [2], and must be distinguished from general fatigue probably affecting ten times as many. A genetic predisposition for ME/CFS has been demonstrated [3].

ME/CFS has profound impact on quality of life for patients and caretakers [4]. The symptom burden is heavy [5], and the disease carries high socioeconomic costs. Patients with severe ME/CFS suffer major functional impairments and often a range of debilitating symptoms. No standard drug treatment has been established, mostly due to lack of knowledge of the underlying disease mechanisms.

We have performed a pilot case series of three patients suggesting clinical activity for B-cell depletion using the monoclonal anti-CD20 antibody rituximab [6]. The case series was followed by a small, randomized, double-blind and placebo-controlled phase II study of 30 patients given either rituximab (two infusions two weeks apart), or placebo, with follow-up for 12 months [7]. The primary endpoint was negative, i.e. there was no difference between the rituximab and placebo groups at 3 months follow-up. There was, however, a significant difference in favor of the rituximab group in the course of *Fatigue score* during follow-up, most evident between 6–10 months follow-up, and with clinical responses in 2/3 of the patients receiving rituximab. The symptom improvements were delayed, starting 2–8 months after initial and rapid B-cell depletion [7], suggesting that ME/CFS in a subgroup of patients could be a variant of an autoimmune disease involving B-lymphocytes and elimination of long-lived antibodies.

According to protocol for the previous randomized KTS-1-2008 study, patients assigned to the placebo group should be given the opportunity to participate in a new open-label study with rituximab. The protocol for the present study was designed to learn about the therapeutic efficacy of rituximab maintenance treatment, for response rates and response durations. Also, the experiences could form the basis for design of a future randomized, double-blind and placebo-controlled trial. Therefore, we have now performed this open-label phase II study (KTS-2-2010) using rituximab induction (two infusions two weeks apart) followed by rituximab maintenance infusions after 3, 6, 10 and 15 months, and with follow-up for three years.

## Materials and Methods

### Ethics

The study, including one amendment, was approved by the Regional Ethical Committee in Norway, no 2010/1318-4, and by the National Medicines Agency. All patients gave written informed consent.

### Study design and pre-treatment evaluation

This study (KTS-2-2010, EudraCT no. 2010-020481-17, ClinicalTrials.gov NCT01156909) was designed as a single center, open-label phase II trial, one-armed with no randomization, comprising 29 patients (including two pilot patients) with ME/CFS. The main aim was to evaluate the effect of rituximab induction and maintenance treatment on response rates and response durations, and any adverse effects of the treatment, within 36 months follow-up, and to gain experience for the purpose of designing a new multicentre, randomized, double-blind, and placebo-controlled trial. The Protocol for this study, including one amendment, is available as supporting information (S1 Protocol).

The inclusion criteria were: a diagnosis of ME/CFS according to the Fukuda 1994 criteria [8], and age 18–66 years. Exclusion criteria were: fatigue not meeting the diagnostic criteria for ME/CFS, pregnancy or lactation, previous malignant disease (except basal cell carcinoma in skin or cervical dysplasia), previous severe immune system disease (except autoimmune diseases such as e.g. thyroiditis or diabetes type I), previous long-term systemic immunosuppressive treatment (such as azathioprine, cyclosporine, mycophenolate mofetil, except steroid courses for e.g. obstructive lung disease), endogenous depression, lack of ability to adhere to protocol, known multi-allergy with clinical risk from rituximab infusions, reduced kidney function (serum creatinine > 1.5x upper normal value), reduced liver function (serum bilirubin or liver transaminases > 1.5x upper normal), known HIV infection, or evidence of ongoing active and relevant infection.

The pre-treatment evaluation included standard laboratory tests (hematology, liver function, renal function), HCG to exclude pregnancy in fertile women, endocrine assessment (thyroid, adrenal, prolactin), serology for virus (EBV, CMV, HSV, VZV, Enterovirus, Parvovirus B19, adenovirus), immunophenotyping of peripheral blood lymphocyte subsets, common autoantibodies, and serum immunoglobulins (IgG, IgM, IgA) with IgG subclasses. MRI of the brain was previously performed in all patients. Further diagnostic tests were performed if the pre-treatment evaluation including clinical examination revealed any relevant abnormality that could explain the severe fatigue experienced by the patients.

### Self-reported ME/CFS symptom scoring

Before intervention, the patients assessed their ME/CFS disease status the last three months and recorded their symptoms according to a scale (1–10; 1: no symptom; 5: moderate symptom; 10: very severe symptom) (S1 Fig).

During follow-up, every second week the patients recorded the overall change in each symptom during the preceding two weeks, always compared to baseline (S2 Fig). The scale (0–6) for the follow-up form was: 0: Major worsening; 1: Moderate worsening; 2: Slight worsening; 3: No change from baseline; 4: Slight improvement; 5: Moderate improvement; 6: Major improvement. These forms for self-reported symptoms were similar to those used in the previous randomized phase II study [7].

The self-reported symptom changes recorded every second week and always compared to baseline are relative, i.e. an improvement interpreted as moderate or major will be different in a patient with severe ME/CFS who is mostly bedridden, and a patient with mild ME/CFS who is able to perform some level of activity. Therefore, the patients also registered their “Function level” on a scale 0–100%, according to a form with examples in which 100% denoted a completely healthy state as before acquiring ME/CFS (S3 Fig). According to this form, patients with very severe ME/CFS will report a Function level < 5%, patients with severe ME/CFS will report Function level 5–10% (mostly bedridden), patients with moderate ME/CFS 10–15% (mainly housebound), and patients with milder degree of ME/CFS a Function level in the range of 20–50%. The patients estimated self-reported Function level at baseline, at 15, 24 and 36 months. This recording of Function level was not predefined in the protocol.

At baseline, and at 3, 6, 10, 15, 20, 24, 30 and 36 months, the patients recorded the SF-36 Norwegian version 1.2 short form scheme, which is a general health-related quality of life questionnaire assessing Physical health summary score, Mental health summary score, and eight subdimensions (Physical function, Role-physical, Bodily pain, Vitality, General health, Social function, Role-emotional, and Mental health) [9,10]. Both norm-based scores based on population mean values of approximately 50, according to population norm US 1998, and raw scores (scale 0–100) were calculated.

### Endpoints

The primary endpoint was effect on self-reported ME/CFS symptoms during follow-up. A clinical response period was defined as *Fatigue score* ≥ 4.5 for at least six consecutive weeks (i.e. for at least three consecutive two-week recordings), which must also include at least one recording of *Fatigue score* > 5.0 during the response period, but not predefined to any specific time interval during three years follow-up. Single response periods, and the sum of such periods were recorded as response durations during follow-up.

Secondary endpoints were effects on the ME/CFS symptoms, at 3, 6, 10, 15, 20, 24, 30 and 36 months assessed by the SF-36 questionnaire, the longest consecutive response period (continuous mean *Fatigue score* ≥ 4.5), the fraction of included patients still in response at end of study (36 months), and toxicity during follow-up.

### Treatment schedule and follow-up

The patients were given rituximab infusions in the outpatient clinic at Department of Oncology, Haukeland University Hospital. The induction treatment, rituximab 500 mg/m^2^ (maximum 1000 mg), diluted in saline to a concentration of 2 mg/ml, was administered twice with two weeks interval, with nurse surveillance and according to local guidelines. The patients then received rituximab maintenance infusions, 500 mg/m^2^ (maximum 1000 mg) at 3, 6, 10 and 15 months follow-up. All patients were given oral cetirizine 10 mg, paracetamol 1 g, and dexamethasone 8 mg prior to infusion. The two pilot patients received only one rituximab induction infusion, with the sixth (last) infusion at 18 and 19 months (instead of 15 months) respectively.

According to protocol, the planned 10 and 15 months infusions could be omitted if there were no signs of clinical response at the 10-months visit.

The present open-label phase II study also had exploratory elements, aiming to gain knowledge on dose-response relationships for proper design of a later randomized phase III study. Therefore, by December 2011 an amendment was submitted to and approved by The Regional Ethical Committee; for patients with slow and gradual improvement after 12 months follow-up including five rituximab infusions, up to six additional rituximab infusions could be given with at least two months intervals. No other intervention should be given during follow-up. After infusions, the patients attended the outpatient clinic at 20, 24, 30 and 36 months, for assessment of the clinical course of their disease, including delivery of self-reported symptom forms. Most of the patients including those still in ongoing remission at the end of follow-up, have been assessed at regular intervals even after the study period.

### Response definitions and statistical analyses

Similar to the previous randomized phase II study [7], the self-reported symptom changes recorded every second week and always compared to baseline (S2 Fig scale 0–6), were used to calculate symptom scores during follow-up. The *Fatigue score* was calculated every second week as the mean of the four symptoms: Fatigue, Post-exertional malaise, Need for rest, Daily functioning. The *Pain score* was calculated as the mean of the two dominating pain symptoms (if pre-treatment level ≥ 4, among Muscle pain, Joint pain, Headache, Cutaneous pain). The *Cognitive score* was the mean of the three symptoms: Concentration ability, Memory disturbance, Mental tiredness. The *Fatigue score*, *Pain score*, and *Cognitive score* were plotted every second week, for each patient in separate diagrams.

The main response was defined from the *Fatigue score*. For overall response rate (ORR) a clinical response was defined as a *Fatigue score* ≥ 4.5 for at least six consecutive weeks, which must include at least one recording of *Fatigue score* > 5.0 during the response period. For each patient, the mean of *Fatigue score*s for the time intervals 0–6, 6–12, 12–18, 18–24, 24–30 and 30–36 months were calculated. For the 28 patients receiving rituximab induction and maintenance treatment, the *Fatigue scores* for the time intervals (mean with 95% confidence intervals) were then plotted.

For the nine patients in this study who had been randomized to the placebo group in the previous phase II study [7], mean *Fatigue scores* for the time intervals 0–3, 3–6, 6–9 and 9–12 months in the present study with rituximab maintenance treatment were plotted together with mean *Fatigue scores* for the same time intervals from their 12 months follow-up in the placebo group in the previous study (in other words, these nine patients were their own “historic controls”). General linear model (GLM) for repeated measures was used to compare the differences in distribution of *Fatigue scores* for the consecutive 3-months time intervals during 12 months follow-up, between the same nine patients when participating in the present rituximab maintenance (KTS-2-2010) study and when participating in the placebo group in KTS-1-2008 study. Mean *Fatigue scores* for four time intervals were included in the analyses, and Greenhouse-Geisser adjustments were made due to significant Mauchly’s tests for sphericity. The main effect for the interaction between time and group (i.e. rituximab maintenance versus “historical” placebo) was assessed.

SF-36 data from baseline, and from 3, 6, 10, 15, 20, 24, 30, and 36 months follow-up were analyzed using a SPSS syntax file, with both raw scores (scale 0–100) and norm-based scores in which the population mean score is approximately 50 (according to US 1998). For analysis of correlation between “SF-36mean5” and self-reported Function level, SF-36 raw scores were used. The statistical analyses were performed using SPSS for Macintosh, ver. 22, and Graphpad Prism ver. 6. Punching of data was performed by ØF and by the staff at the office for cancer research at the Dept. of Oncology. Analyses were performed by ØF and OM. Accuracy of data punching and analyses were checked by IGR and KS.

### Patient characteristics

**Fig 1:**
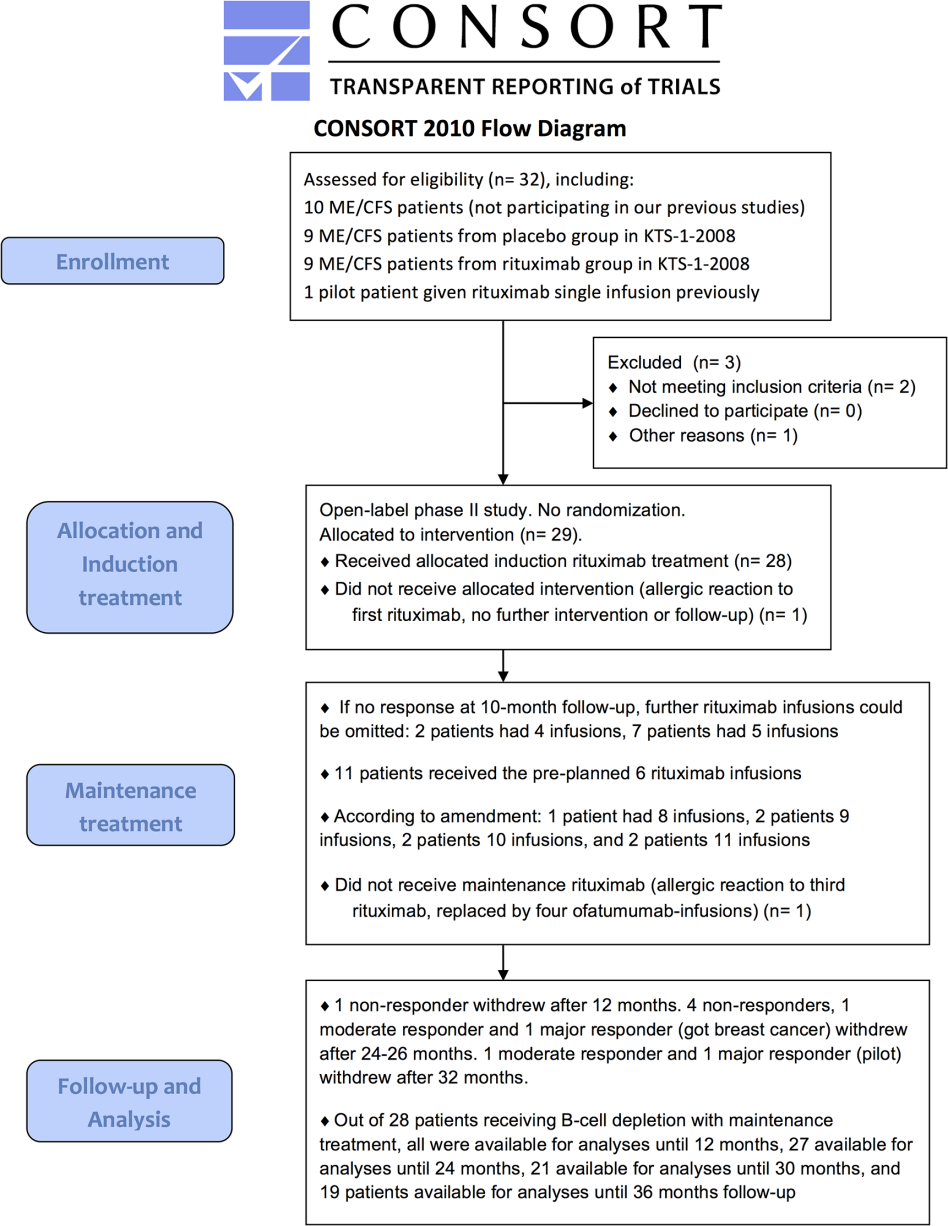
Consort 2010 Flow Diagram for the KTS-2-2010 study. Consort Flow Diagram for the KTS-2-2010 study, with enrollment, allocation to induction and maintenance rituximab treatment, and follow-up showing number of patients who withdrew from study before 36 months.

The Consort flow diagram for the KTS-2-2010 study is shown in Fig 1. Between September 2010 and February 2011, 27 patients were included. In addition, two pilot patients started maintenance rituximab treatment from July 2009. End of follow-up was in February 2014. The patients were partly recruited through referrals from the Dept. of Neurology, Haukeland University Hospital, and partly through primary care physicians.

Among the 29 patients, 20 (69%) were women. The mean age was 40 years (range 21–59 years). The mean duration of ME/CFS was 9 years (range 1–20 years). Out of 29 included patients, 13 had moderate severity (mainly housebound), four had moderate/severe, and three had severe (mainly bedridden) ME/CFS. Four had mild/moderate and five patients mild ME/CFS [11] (Table 1). During the preceding year before inclusion, 17 patients reported a stable ME/CFS disease, five had experienced worsening of symptoms, and seven had relapse after previous rituximab-associated responses. Infection before ME/CFS onset had been evident in 17 patients (59%), in two patients (7%) a relation to preceding infection was possible, and there was no clear history of infection upfront in 10 (34%).

**Table 1:** ME/CFS disease characteristics and selected response data, for 29 patients included in the study.

Supp. Fig.no, CS^1^	Sex, Age^2^	Infection upfront^3^	ME/CFS duration, severity^4^	Previous study^5^	Family AD^6^	Rx infus^7^	Clinical response^8^ (weeks)	Response duration^9^ (weeks)	Response at study end^10^	Notes^11^	SF-36mean5 raw score^12^, at 0-15-20/24-36m	Function level^13^ at 0-15-24-36m
**S4A MajR**	F, 24	Yes, Mono	8 y, mod	Pilot, Rx,R-Re	No	6 Rx	22–156	134	Yes, 36m	LON 34m	36-nd-99-99	10-100-100-100
**S4B MajR**	F, 46	Possible	13 y, mod	KTS-1, Rx,R-Re	Yes	6 Rx	16–66, 78–156	128	Yes, 36m	Rx-Wor x1	32-100-100-100	5-100-100-100
**S4C MajR**	F, 25	Yes, Mono	11 y, mod	KTS-1, Pl,NoR	No	5 Rx	16–156	140	Yes, 36m	Airw.inf.	29-77-76-81	20-85-95-95
**S4D MajR**	M, 21	Yes, Viral	8 y, mild	KTS-1, Pl,NoR	No	6 Rx	16–156	140	Yes, 36m		32-59-79-100	35-90-95-95
**S4E MajR**	F, 28	No	12 y, mod	KTS-1, Pl,NoR	No	6 Rx	12–156	144	Yes, 36m, SR	LON 22m	38-89-92-74	25-90-100-85
**S4F MajR**	F, 38	No	20 y, mild	KTS-1, Rx,R-Re	No	6 Rx	34–46, 50–156	118	Yes, 36m	Rx-Wor x3	38-63-72-78	15-70-80-90
**S4G MajR**	M, 26	Yes Mono	8 y, mild/mod	No	Yes	6 Rx	22–44, 46–70, 74–156	128	Yes, 36m	Rx-Wor x2	39-81-83-82	25-85-85-85
**S4H MajR**	F, 56	Yes, Pneum.	11 y, mild/mod	No	No	6 Rx	8–24, 38–44, 48–104, 130–136	84	No, 36m		27-81-92-32	10-85-85-25
**S4I MajR**	M, 59	No	3 y, mod/sev	No	Yes	6 Rx	36–48, 60–144	96	No, 36m	Rx-Wor x1	15-61-61-25	5-60-60-30
**S4J MajR**	F, 42	Yes, GI	7 y, sev	No	No	2 Rx 4 Of	14–24, 50–60, 64–88	44	No, OoSt 24m	Allergy,BC 24m	17-58-72-nd	5-60-75-nd
**S4K MajR**	F, 38	No	10 y, mild	KTS-1, Rx,NoR	No	6 Rx	20–26, 32–44, 52–72, 78–112	72	No, 36m	Rx-Wor x3	15-68-78-21	15-75-85-25
**S4L MajR**	F, 39	Yes, Airway	7 y, mod/sev	KTS-1*, Rx,R-Re	No	6 Rx	18–24, 28–42, 50–64, 70–86, 92–130	88	No, 32m OoSt 32m	Rx-Wor x6	nd-nd-nd-nd	10-90-90-nd
**S5M MajR**	F, 23	No	5 y, mod	KTS-1, Rx,R-Re	No	11 Rx	26–36, 38–60, 84–100, 114–156	90	Yes, 36m		36-38-64-88	10-75-75-70
**S5N MajR**	M, 49	No	12 y, mod	KTS-1, Pl,NoR	Yes	9 Rx	94–156	62	Yes, 36m		38-35-44-60	10-50-70-72
**S5O ModR**	F, 47	Yes, Mono	4 y, mod	No	Yes	10 Rx	28–42, 46–54, 66–88, 92–144	96	No, 33m	Rx-Wor x3	28-37-40-35	7-18-30-31
**S5P ModR**	M, 58	No	17 y, mod	KTS-1, Pl,NoR	Yes	9 Rx	58–112	54*	Yes, 25m OoSt 25m		35-52-52-nd	15-40-40-nd
**S5Q ModR**	F, 44	Yes, GI	5 y, sev	KTS-1, Pl,NoR	No	10 Rx	64–80, 82–94, 96–102, 112–124	46	No, 32m OoSt 32m	Rx-Wor x3	14-30-60-12	5-70-70-20
**S5R ModR**	M, 50	Possible, Airway	17 y, mod/sev	KTS-1, Pl,NoR	Yes	11 Rx	72–142, 148–156	78	Yes, 36m	Gallstone 33m	19-26-32-32	8-19-32-38
**S6U MargR**	F, 56	Yes, Mono	8 y, mod	KTS-1, Rx,NoR	Yes	5 Rx	76–92	16**	No, 36m	No sign. response	26-23-33-29	13-17-75-15
**S6S NonR**	F, 42	Yes, Borrelia	5 y, mild	KTS-1, Rx,R-Re	No	4 Rx	No response	0	No, 35m		33-22-35-58	20-25-35-35
**S6T NonR**	F, 21	Yes, Airway	7 y, mod	KTS-1, Rx,R-Re	Yes	8 Rx	No response	0	No, OoSt 24m		45-nd-41-nd	20-65-65-nd
**S6V NonR**	M, 50	No	1 y, mild	No	No	5 Rx	No response	0	No, 36m	*See comment	21-43-51-44	50-70-75-75
**S6W NonR**	F, 26	Yes, GI	3 y, mild/mod	KTS-1, Pl,NoR	Yes	5 Rx	No response	0	No, 36m	Airw.inf, ITP 36m	21-32-30-30	15-20-20-20
**S6X NonR**	F, 37	No	20 y, mod	KTS-1, Pl,NoR	Yes	5 Rx	No response	0	No, OoSt 24m		23-21-14-nd	15-15-15-nd
**S6Y NonR**	F, 32	No	9 y, mod/sev	No	Yes	6 Rx	No response	0	No, OoSt 24m		20-19-19-nd	8-8-12-nd
**S6Z1 NonR**	M, 51	Yes, GI	8 y, mild/mod	KTS-1, Rx,R-Re	No	4 Rx	No response	0	No, OoSt 12m	MargR KTS-1	33-33-nd-nd (0m and 12m)	20-22-nd-nd (0m and 12m)
**S6Z2 NonR**	F, 49	Yes, Mono	13 y, mod	No	No	5 Rx	No response	0	No, OoSt 24m		30-26-36-nd	15-15-15-nd
**S6Z3 NonR**	F, 56	Yes, Viral	5 y, sev	No	No	5 Rx	No response	0	No, 36m		7-9-11-6	8-6-4-3
*See legend	M, 35	Yes, Throat	1.5 y, mod	No	No	1 Rx	nd		OoSt 0m*	Allergy	nd	nd

^1^: The numbers refer to corresponding plots in S4, S5 and S6 Figs (i.e. S4A means S4 Fig panel A) for each of 28 individual patients receiving rituximab maintenance treatment. Among 18 patients fulfilling the predefined response criterion, clinical significance (CS) was determined post-hoc as Major (MajR, n = 14) or Moderate (ModR, n = 4). One patient had a marginal response (MargR) and nine patients were non-responders (NonR).

*: one patient (last row in Table 1) had an allergic reaction to the first rituximab infusion and did not receive induction treatment or complete follow-up.

^2^: F: female, M: male. Age in years.

^3^: Indicating infection preceding ME/CFS onset. Mono: mononucleosis; Viral: viral infection not otherwise specified; GI: gastrointestinal infection; Airway: upper airways infection; Borrelia: Borrelia infection; Pneum.: pneumonia.

^4^: ME/CFS disease duration given in years (y). ME/CFS severity categorized as mild: mild grade; mild/mod: mild to moderate grade; mod: moderate grade, mod/sev: moderate to severe grade; sev: severe grade (see Materials and Methods).

^5^: Previous participation in clinical studies on ME/CFS. Pilot: pilot patient. KTS-1: participated in the KTS-1-2008 study; Pl,NoR: previously given placebo in KTS-1-2008 with no clinical response; Rx,R-Re: previously given rituximab in KTS-1-2008 (or as pilot) with clinical response and later relapse; Rx,NoR: previously given rituximab in KTS-1-2008 with no clinical response; No: the patient had not participated in previous studies. *: this patient (corresponding to S4 Fig panel L) was a participant in KTS-1-2008 and also a pilot patient for this study.

^6^: Autoimmune diseases (AD) among first-degree relatives were present for 12 out of 29 included patients (41%). These AD included rheumatoid arthritis (in relatives of seven patients), Sjøgren’s syndrome (in relatives of two patients), ulcerative colitis (in relatives of three patients), thyroiditis (in relatives of two patients). Also, glomerulonephritis, lupus, juvenile arthritis, psoriasis, multiple sclerosis (all present in relatives of single patients).

^7^: Rx infus: number of rituximab (Rx) infusions given to each patient (see Materials and Methods, and Results).

^8^: Clinical response periods according to predefined criteria, i.e. periods of *Fatigue score* ≥ 4.5 for at least six consecutive weeks which must include at least one recording of *Fatigue score* >5.0 during the response period. The numbers show the intervals with clinical response given in weeks during follow-up.

^9^: Response durations defined as the sum of individual clinical response periods during follow-up, and given in weeks. *: This patient had a moderate improvement from 58–112 weeks, and with a mean *Fatigue score* exactly 5.0 in the interval 80–112 weeks. He did not fulfill strict response criteria, but was post-hoc judged as a moderate responder.

**: This patient fulfilled predefined response criteria, but had a short lasting and late response period, interpreted post-hoc as probably not related to intervention and not clinically significant.

^10^: Clinical response status at end of study. Also shown the time of last recording for each patient. Yes: still in clinical response at end of follow-up; No: not in clinical response at end of follow-up; OoSt: out of study, also indicating time point during follow-up; SR: slight symptom increase but still in response;

*: allergic reaction to first rituximab infusion, did not complete induction therapy or follow-up.

^11^: LON: late onset neutropenia; Airw.inf.: several airways infections; Rx-Wor: indicating number of transient worsening of ME/CFS-symptoms after rituximab infusions; Allergy: allergic reaction to rituximab; BC: breast cancer; ITP: idiopathic thrombocytopenic purpura (also indicated in the corresponding panels in S4, S5 and S6 Figs);

*See comment: this patient had improvement during follow-up assessed by SF-36 scores and selfreported Function level, but did not fulfill criteria for clinical response; MargR KTS-1: this patient had a marginal response duration in the previous KTS-1-2008 study; No sign. response: not interpreted as a clinically significant response.

^12^: SF-36mean5 is the mean of raw scores for the five SF-36 subdimentions Physical function, Bodily pain, Vitality, General health, and Social function (scales 0–100), shown at 0, 15, 20 or 24, and 36 months follow-up (in three patients last recording at 31, 33 and 35 months, respectively); nd: not done.

^13^: Self-reported Function level, given as per cent (0–100%) in which 100% denotes completely healthy, according to a form with examples (S3 Fig), recorded at 0, 15, 24 and 36 months follow-up (in three patients last recording at 31, 33 and 35 months, respectively); nd: not done.

In three patients (10%) an autoimmune disease had previously been diagnosed (thyroiditis, psoriasis, juvenile arthritis). In 12 patients (41%) an autoimmune disease had been diagnosed among first-degree relatives (Table 1). In addition, three patients (10%) reported a diagnosis of ME/CFS, and two patients (7%) a diagnosis of fibromyalgia, among their first-degree relatives.

Nine patients from the rituximab group in the KTS-1-2008 study, and one pilot patient who had received a single rituximab infusion previously, were included (Table 1, Fig 1). Among these ten patients, two had no response to rituximab during the previous KTS-1-2008 study, one had a marginal response, and seven had clinical response but subsequent relapse of ME/CFS before entering the present KTS-2-2010 study. Nine patients from the placebo group in the randomized KTS-1-2008 study were included in the present study (Table 1, Fig 1). Thus, out of 29 included, 10 patients (34%) had not participated in our previous clinical studies for ME/CFS.

The baseline symptom scores derived from the baseline forms (scale 1–10, S1 Fig), self-reported baseline Function level (scale 0–100%, form with examples in S3 Fig), and baseline SF-36 norm-based scores, are shown in Table 2.

**Table 2:** Baseline self-reported symptom scores, baseline Function level, and baseline norm-based SF-36 scores, for 28 patients receiving rituximab induction and maintenance treatment.

	All patients	Responders^a^	Non-responders^b^
**Baseline symptom scores** ^c^	n = 28	n = 18	n = 10
Fatigue score^d^ mean (range)	8.0 (6.3–10.0)	8.0 (6.5–9.3)	8.0 (6.3–10.0)
Cognitive score^e^	7.5 (4.7–10.0)	7.3 (4.7–8.7)	7.9 (5.7–10.0)
Pain score^f^	7.2 (4.0–9.0)	7.2 (4.0–9.0)	7.4 (5.5–9.0)
ME/CFS overall score^g^	8.2 (6.0–10.0)	8.0 (6.0–9.0)	8.4 (6.0–10.0)
**Baseline Function level (%)** ^h^	n = 28	n = 18	n = 10
mean (range)	15.0 (5.0–50.0)	13.1 (5.0–35.0)	18.6 (8.0–50.0)
**Baseline SF-36, norm-based** ^i^	n = 27	n = 17	n = 10
Physical Health Summary, mean (SD)	25.6 (6.6)	27.0 (7.2)	23.2 (5.1)
Mental Health Summary	44.6 (10.4)	43.3 (10.5)	46.7 (10.4)
Physical Function	33.0 (7.9)	34.0 (8.4)	31.3 (6.9)
Role Physical	29.1 (3.4)	29.4 (4.0)	28.5 (2.2)
Bodily Pain	32.0 (8.4)	33.0 (8.9)	30.4 (7.7)
Vitality	31.6 (5.9)	31.7 (6.1)	31.6 (5.9)
General Health	31.1 (5.0)	32.0 (5.6)	29.5 (3.5)
Social Function	21.4 (8.7)	20.6 (8.7)	22.9 (9.1)
Role Emotional	48.9 (11.1)	48.8 (11.5)	49.2 (11.0)
Mental Health	46.9 (10.7)	45.6 (9.7)	48.9 (12.5)

^a^: Clinically significant responders, including 14 major and four moderate responders.

^b^: Patients with no clinically significant response, including one patient with a marginal response and nine non-responders.

^c^: Baseline self-reported symptom scores assessed for the preceding 3-months period before start of intervention (scale 1–10; 1: no symptom, 5: moderate symptom, 10: very severe symptom, see S1 Fig).

^d^: Fatigue score was the mean score for the four symptoms: Fatigue, Malaise after exertion, Need for rest, Daily functioning.

^e^: Cognitive score was the mean score for the three symptoms: Concentration ability, Memory disturbance, Mental tiredness.

^f^: Pain score was the mean score for the two dominating pain symptoms, from Muscle pain, Headache, Joint pain, Cutaneous pain (if pretreatment score at least 4).

^g^: ME/CFS overall score was the patient’s interpretation of overall symptom burden at baseline.

^h^: Baseline self-reported Function level (scale 0–100%, in which 100% denotes a completely healthy state), according to a form with examples (see S3 Fig).

^i^: SF-36 norm-based scores at baseline (population mean approximately 50).

### B-lymphocytes

Lymphocyte subpopulations, including CD19 positive B-cells, were determined in EDTA anticoagulated blood samples before treatment, and during all follow-up visits (3, 6, 10, 15, 20, 24, 30 and 36 months). Immunophenotyping of lymphocyte subpopulations was performed using the BD Multitest 6-color TBNK kit with BD Trucount Tubes for relative and absolute concentration determination (BD Biosciences). The samples were prepared according to the manufacturer’s instructions and immediately analyzed on a BD Canto II flowcytometer (BD Biosciences). Immunoglobulin levels in serum (IgG, IgA and IgM) were measured at all visits.

## Results

### Inclusion, treatment schedules and follow-up

A total of 29 patients, including two pilot patients, met the Fukuda criteria [8] and were accepted for the KTS-2-2010 study. All 29 patients also fulfilled the Canadian diagnostic criteria (2003) for ME/CFS [1]. The latter identify patients with more severe symptoms and more functional impairment than patients identified only by Fukuda criteria [12].

Two patients received four, and seven patients received five rituximab infusions including one major responder in whom the sixth infusion was omitted due to upper airways infections (Table 1, S4 Fig panel C). Eleven patients received the planned six rituximab-infusions. According to the amendment, seven patients with ongoing slow and gradual improvement of ME/CFS symptoms after 12 months received additional rituximab infusions; one patient received eight, two patients received nine, two patients received ten, and two patients received in total 11 rituximab infusions (Table 1).

One patient experienced dyspnea interpreted as an allergic reaction during the first rituximab-infusion; his symptoms gradually declined and normalized within weeks. Two weeks after the infusion his X-ray, lung function and gas diffusion tests were normal. He did not receive further B-cell depletion, and as such he failed to complete the induction treatment. One patient had a distinct allergic reaction including an urticarial exanthema at the end of the 3-months rituximab infusion. She then had a major transient clinical response between 3 and 6 months follow-up. Due to a ME/CFS symptom relapse she was subsequently treated with the humanized monoclonal anti-CD20 antibody ofatumumab [13,14] from 8 months follow-up, with no allergic reaction and again with a clear clinical response starting three months later (from 11 months follow-up). This patient received a total of four ofatumumab infusions (Table 1, S4 Fig panel J), but withdrew from study after 24 months because she was diagnosed with breast cancer.

One patient with no response decided to withdraw from follow-up after 12 months (Table 1, S6 Fig panel Z1). Four other non-responders decided to withdraw after approximately 24 months (Table 1, S6 Fig panels T,X,Y,Z2) because they wanted to try alternative interventions. One moderate responder withdrew after 25 months (Table 1, S5 Fig panel P), one moderate responder withdrew after 32 months (Table 1, S5 Fig panel Q) due to increasing symptoms interpreted by the patient as early signs of a possible relapse (which turned out not to be the case), and one major responder (pilot) withdrew after 32 months after experiencing a full ME/CFS relapse (Table 1, S4 Fig panel L).

### Clinical responses

In this study, according to predefined response criteria, 18 patients out of the 29 included (intention to treat) had self-reported *Fatigue score* ≥ 4.5 for at least six consecutive weeks, including at least one recording of *Fatigue score* > 5.0 during the response period, giving an overall response rate of 62% (95%CI 44%–77%).

This open-label phase II study also had exploratory elements, aiming to elucidate the dose-response relationships and to aid the design of a new randomized phase III study. Clinically significant response, determined at the end of study, was seen in 18 out of 28 patients receiving rituximab induction and maintenance treatment (64%, 95%CI 46%–79%).

**Fig 2:**
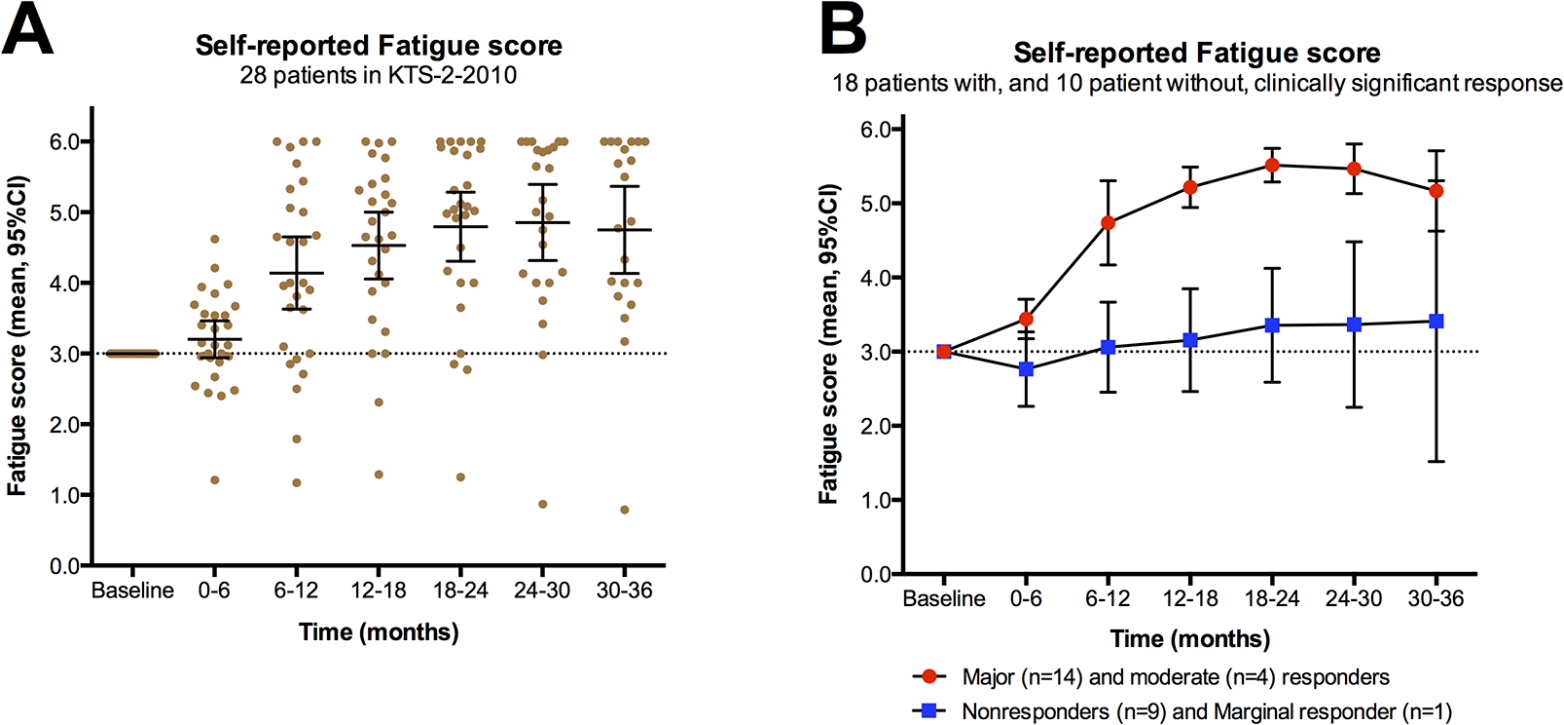
Self-reported Fatigue scores for 28 patients receiving rituximab induction and maintenance treatment. *Fatigue score* was recorded every second week, always compared to baseline, as the mean of four fatigue-related symptoms (scale 0–6; 3: no change from baseline; 4, 5, 6: slight, moderate, major improvement, respectively; 2, 1, 0: slight, moderate, major worsening, respectively). Panel A shows *Fatigue scores* for the time intervals 0–6, 6–12, 12–18, 18–24, 24–30 and 30–36 months, with means and 95% CI for each time interval. In panel B the corresponding *Fatigue scores* are shown for each time interval during follow-up, divided between 18 patients with clinically significant responses, and 10 patients with either marginal response (n = 1) or no response (n = 9). Out of 10 patients with no clinically significant response, one patient withdrew from study after 12 months, and four patients after 24–26 months follow-up. Out of 18 patients with clinically significant responses, one withdrew from study after 24 months due to a diagnosis of T2N0 breast cancer, two moderate responders withdrew after 25 and 32 months, respectively, and one major responder withdrew after 32 months.

The self-reported *Fatigue scores* for the 28 patients given rituximab induction and maintenance treatment are shown in Fig 2A, with mean *Fatigue scores* for each six-months time interval during three years follow-up. Table 1 shows data for each of these 28 patients, including sex, age, possible preceding infection, ME/CFS disease characteristics (duration and severity), participation in previous studies, autoimmunity among first-degree relatives, number of rituximab infusions, response periods and response durations during follow-up, clinical status at end of study, and also SF-36 and Function level data at selected time points (0-15-24-36 months).

Data describing courses during follow-up for *Fatigue score*, *Cognitive score*, *Pain score*, together with SF-36 scores and Function level recorded at baseline, and at 15, 24 and 36 months, are also shown for each individual patient in Supporting information (S4 Fig, S5 Fig, S6 Fig).

All patients in the study were followed by ØF and OM. At the end of study, these authors specified post-hoc the clinical significance after assessing the patients through follow-up. This was done to differentiate among the responses, and was not specified in the predefined protocol.

Among the 18 patients with clinically significant responses, 14 were recorded as major responders (Table 1, S4 Fig panels A-L, S5 Fig panels M,N), and four patients as moderate responders (Table 1, S5 Fig panels O-R).

One patient fulfilled the criteria for overall response, but had short response duration, which also occurred late during follow-up (Table 1, S6 Fig panel U). This patient was described as a “marginal” responder and the researchers interpreted her improvement as possibly not related to rituximab intervention and not as a clinically significant response. Contrary, one patient had a long lasting clear but moderate clinical improvement from 58–112 weeks and with *Fatigue score* exactly 5.0 continuously in the interval 80–112 weeks (Table 1, S5 Fig panel P). He did not fulfill strict criteria for response (due to lack of *Fatigue score* >5.0 during the response period). However, based on the post-hoc assessment for each patient, he was yet judged as a moderate responder. Nine patients were classified as non-responders not fulfilling the predefined criteria for response (Table 1, S6 Fig panels S,T,V-Z3).

Fig 2B shows self-reported *Fatigue scores* during follow-up, separate for the 18 patients with clinically significant responses (14 major and 4 moderate), and for 10 patients with either no response (n = 9) or a marginal response (n = 1).

**Fig 3:**
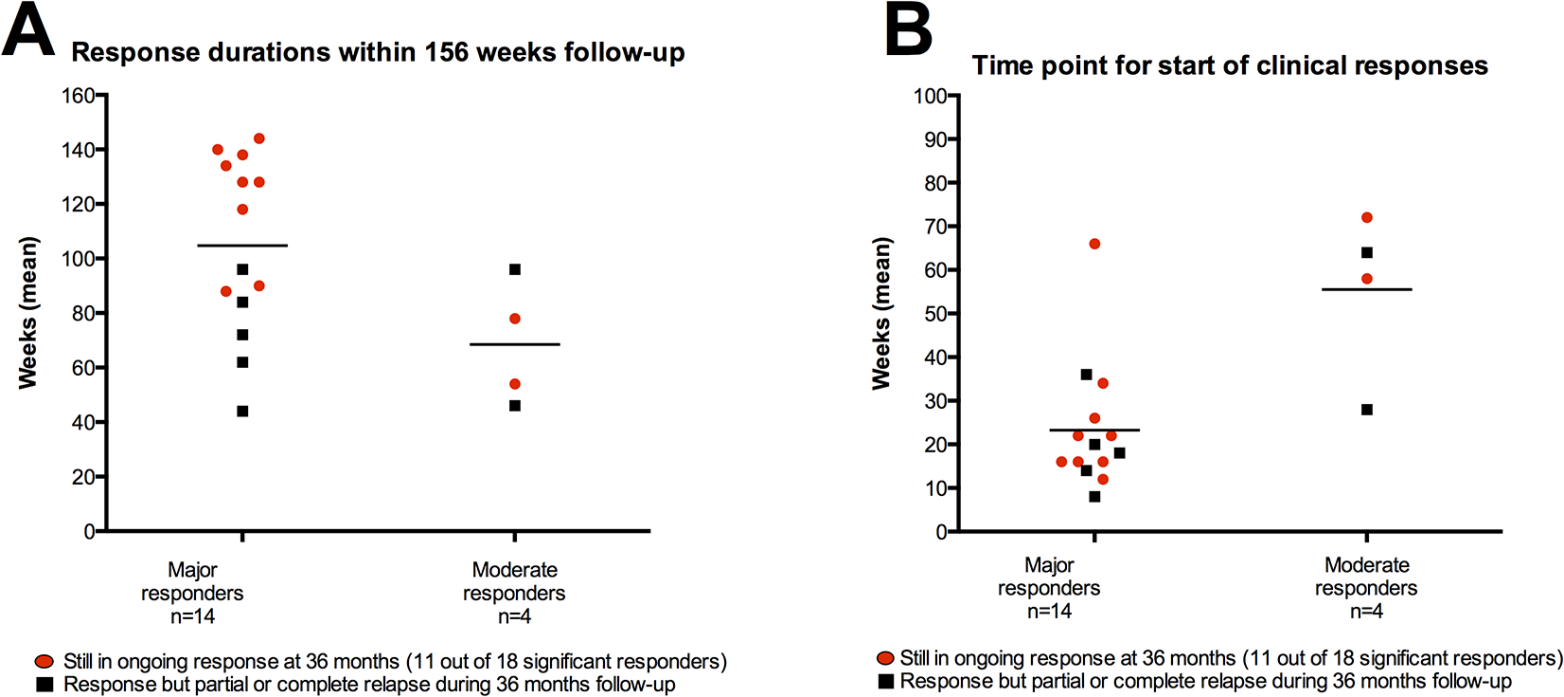
Clinical response durations after rituximab maintenance therapy, within 36 months follow-up. In panel A, response durations within the three years (156 weeks) follow-up are shown, for 14 major responders and four moderate responders. In panel B, time points for *start* of clinical responses are shown, for major and moderate responders. In both panels A and B, the 11 red dots denote patients with still ongoing clinical response at 156 weeks (end of study), while the 7 black squares denote patients experiencing partial or full relapse during the 36 months follow-up period. The overall response criterion was a *Fatigue score* ≥ 4.5 for a minimum of six consecutive weeks, which must include at least one recording of *Fatigue score* > 5.0 during the response period. Single response periods and the sum of response periods during follow-up were recorded as response duration.

For major responders the mean of sum of response durations was 105 weeks, and for moderate responders 69 weeks (Fig 3A) within the 156 weeks study period. For 18 patients with post-hoc defined clinically significant responses the mean of sum of response durations within 156 weeks follow-up was 97 weeks (SD 32 weeks). The mean duration of the longest continuous response (i.e. continuous *Fatigue score* ≥ 4.5) was 83 weeks for major responders, and 48 weeks for moderate responders.

The point of time when clinical responses started was recorded. For the group of major responders, the mean lag time from start of rituximab infusions until start of clinical response was 23 weeks (range 8–66) (Fig 3B). For the four patients with moderate responses, the mean lag time until start of clinical response was 56 weeks (range 28–72) (Fig 3B).

Among 14 patients with major response, at end of study (36 months follow-up, last patient in February 2014) nine were still in ongoing, stable clinical responses (Table 1, Fig 3, S4 Fig panels A-G, S5 Fig panels M,N). At end of follow-up, four patients had experienced different levels of relapse (Table 1, S4 Fig panels H,I,K,L). In addition, one patient was diagnosed with a T2N0 breast cancer at 24 months follow-up. At that time she had slight symptom worsening but not a clear relapse of ME/CFS, subsequent to a response period following ofatumumab infusions (Table 1, S4 Fig panel J).

Among the four moderate responders, two were still in ongoing response at end of follow-up (Table 1); one when he chose to withdraw from study after 25 months (S5 Fig panel P) and one at the end of follow-up at 36 months (S5 Fig panel R). Two patients with moderate response experienced some symptom worsening before end of study. One of these chose to withdraw from the study after 32 months, however the worsening was transient and she remained in moderate response after end of follow-up (Table 1, S5 Fig panel Q). In one a partial relapse occurred from 32 months (Table 1, S5 Fig panel O).

Of the two pilot patients, one experienced a full relapse at approximately 30 months, 11 months after the last rituximab infusion (Table 1, S4 Fig panel L). One pilot patient is still in complete response with no ME/CFS symptoms even after vigorous exercise, five years after the first, and 3 ½ years after the last rituximab infusion (Table 1, S4 Fig panel A).

### Clinical responses in patients from the KTS-1-2008 study

**Fig 4:**
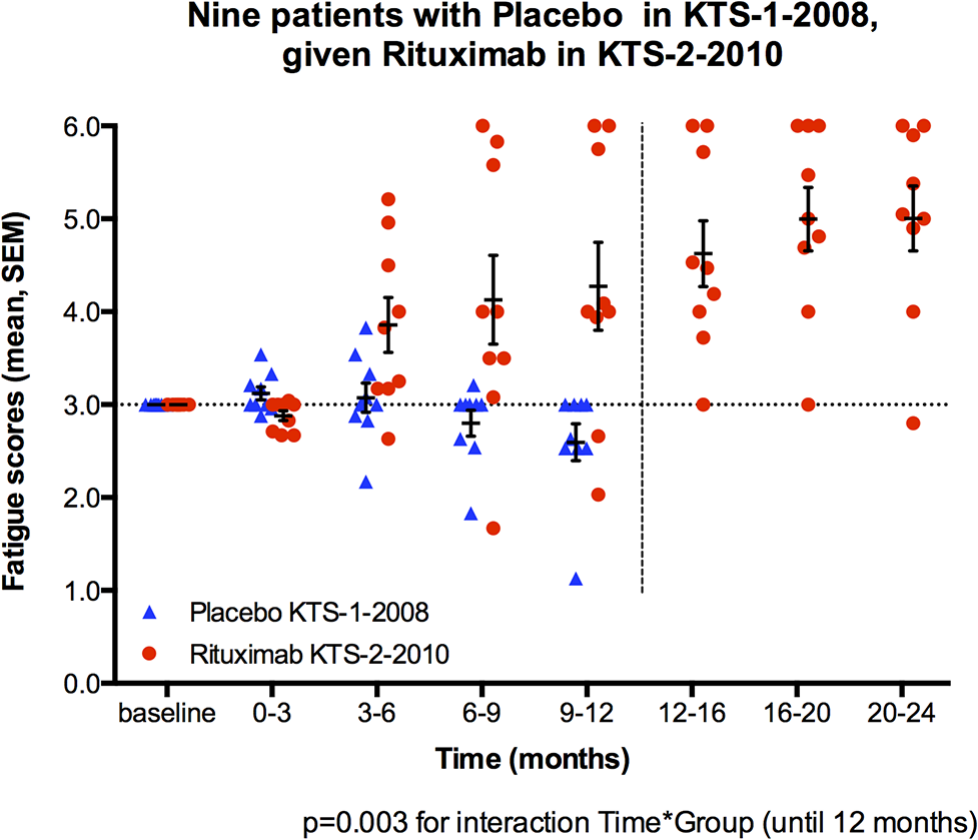
Historic comparison of Fatigue scores for nine patients given placebo in KTS-1-2008, and rituximab maintenance in KTS-2-2010. Nine patients from the placebo group in the previous randomized KTS-1-2008 study were included in the present KTS-2-2010 study with rituximab induction and maintenance treatment. The mean *Fatigue scores* for consecutive 3-months intervals, until 12 months follow-up, were compared using General Linear Model (GLM) for repeated measures. Four time intervals with mean *Fatigue scores* in each were included in the comparison. Main effect for the interaction between time and intervention group (rituximab maintenance versus the patient’s own “historic” placebo) was assessed.

Nine patients from the placebo group in the previous randomized phase II study KTS-1-2008 [7] were included in the present open-label phase II study (KTS-2-2010) with rituximab induction and maintenance infusions (Table 1). None of these nine patients had experienced a clinical response during 12 months follow-up after two placebo infusions two weeks apart. Six out of these nine had clinical response during the first 12 months after rituximab infusions in the present study, and in addition one patient with gradual improvement fulfilled the criteria for a clinical response after 12 months follow-up (Table 1). The *Fatigue scores* for these nine patients, with means for consecutive three-months intervals until 12 months follow-up are shown in Fig 4. There was a significant interaction between time and intervention group (p = 0.003), i.e. there was a difference in course of *Fatigue scores* until 12 months follow-up in favor of rituximab-intervention as compared to “historic” data when the same patients were given placebo.

Nine patients from the rituximab group in the previous KTS-1-2008, and in addition one pilot patient given a single rituximab infusion previously, participated in the present study (Table 1). Among these 10 patients, three had no clinically significant response after rituximab (two infusions two weeks apart) in KTS-1-2008, these were included to explore if prolonged B-cell depletion with maintenance rituximab could result in benefit as compared to induction treatment alone. One of these three non-responders in KTS-1-2008 turned out to be a major responder in the present KTS-2-2010 study, however with a subsequent relapse starting from 27 months follow-up (S4 Fig panel K). One non-responder in KTS-1-2008 was recorded as a “marginal” responder in the present study (S6 Fig panel U) interpreted as probably not related to the rituximab intervention (i.e. the response was short-lasting, occurred late and was not clinically significant). The third patient with no clinically significant response after rituximab in KTS-1-2008 was still a non-responder after rituximab maintenance treatment (S6 Fig panel Z1).

Among the remaining seven patients previously given rituximab with clinical response and subsequent relapse, five were classified as major responders in the present study (Table 1), with significantly prolonged response durations when given rituximab maintenance as compared to their responses after two rituximab infusions as participants in previous KTS-1-2008 (S4 Fig panels B,F,L, S5 Fig panel M), or as compared to the previous response after a single rituximab infusion (S4 Fig panel A). Two patients with relatively short response durations in KTS-1-2008 were classified as non-responders in this study even though they reported slight improvement during follow-up (Table 1, S6 Fig panels S,T).

### Clinical responses after additional rituximab-infusions

Seven patients with slow and gradual improvements of ME/CFS symptoms after 12 months were given further rituximab infusions, according to an approved amendment. Two of these were classified as major responders (Table 1, S5 Fig panels M,N), four remained moderate responders throughout the study in spite of the prolonged B-cell depletion period using extra rituximab infusions (Table 1, S5 Fig panels O-R), while one had no significant response (S6 Fig panel T).

### SF-36 questionnaire on health related quality of life

**Fig 5:**
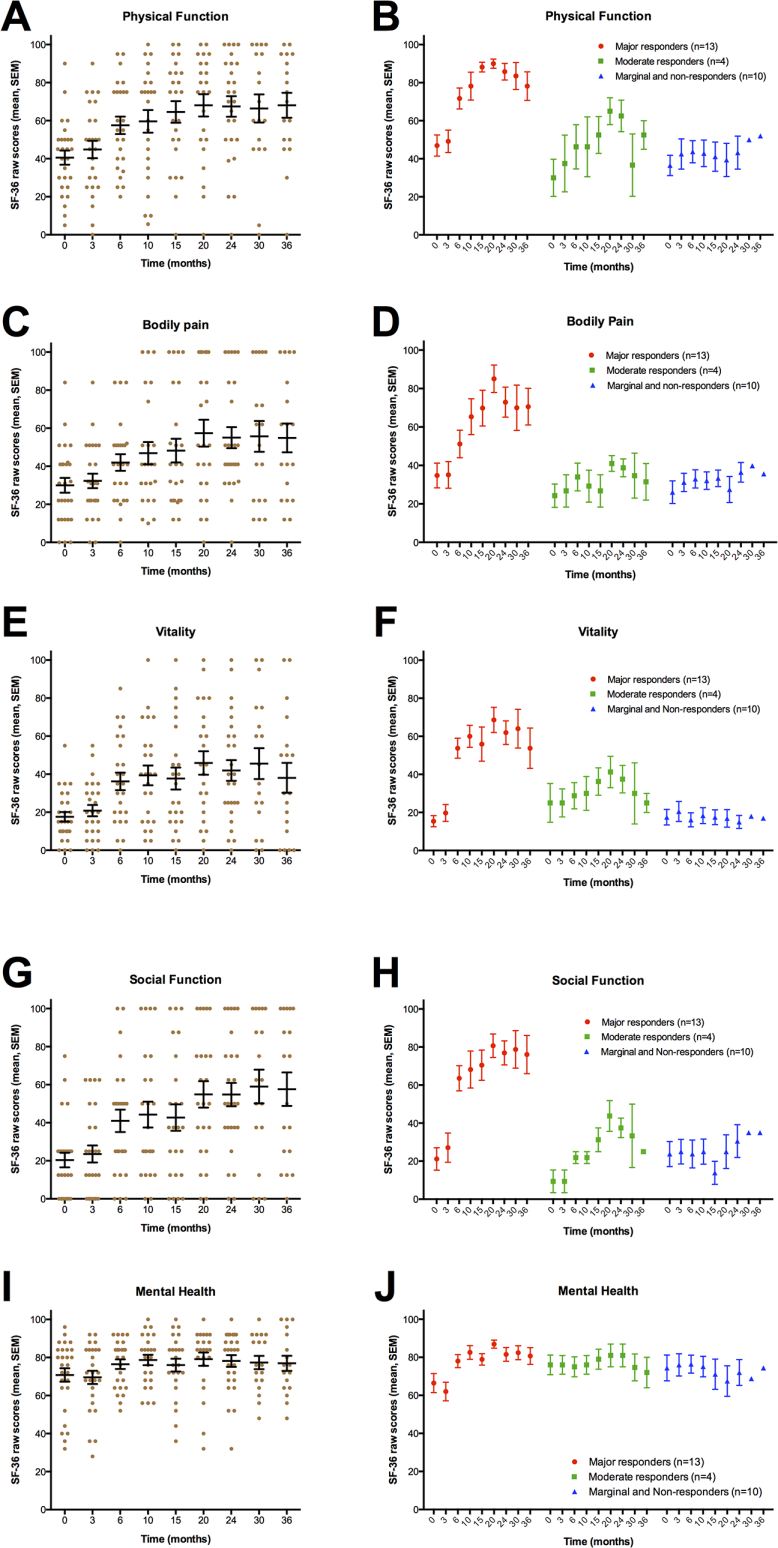
SF-36 questionnaire, raw scores. SF-36 (Norwegian ver. 1.2) forms were recorded at baseline and at 3, 6, 10, 15, 20, 24, 30 and 36 months. SF-36 raw scores (mean, SEM) are shown for 27 patients, for the subdimensions Physical function (panel A), Bodily pain (panel C), Vitality (panel E), Social function (panel G) and Mental health (panel I). In panels B, D, F, H and J are shown the corresponding SF-36 raw scores separately for 13 major responders, four moderate responders, and 10 patients with no clinical significant response (one marginal responder and nine non-responders). One pilot patient (major responder, withdrew from study after 32 months) did not fill in SF-36 forms. One included patient did not receive induction rituximab infusions due to an allergic reaction to the first infusion, and did not fill in SF-36 forms. One major responder was withdrawn from study after 24 months due to being diagnosed with a T2N0 breast cancer. Out of four moderate responders, one withdrew from the study after 25 months, and one after 32 months. Out of 10 patients with no clinically significant response one withdrew from study after 12 months, and four patients after approximately 24 months follow-up.

The SF-36 (Norwegian ver.1.2) questionnaire registering health related quality of life, was recorded by the patients at baseline and at 3, 6, 10, 15, 20, 24, 30 and 36 months follow-up. One pilot patient (major responder) did not fill in SF-36 forms, leaving 27 patients for analysis among those 28 patients who received rituximab maintenance treatment. SF-36 raw scores (scale 0–100) for the subdimensions Physical function, Bodily pain, Vitality, Social function and Mental health are shown in Fig 5, panels A,C,E,G,I for all 27 patients, and in Fig 5, panels B,D,F,H,J with separate data for major responders (n = 13), moderate responders (n = 4), and marginal and non-responders (n = 10). The corresponding norm-based data (according to US 1998 norm) are shown in S7 Fig, in which the horizontal line in each plot denotes the approximate mean population value of 50.

Norm-based and raw scores (mean, SD) for all patients through follow-up are shown in Table 3. In addition, Table 4 shows SF-36 raw scores for the subdimensions Physical function, Bodily pain, General health, Vitality, Social Function, Mental health, and SF-36mean5, in 17 patients with clinically significant response versus 10 patients with no significant response.

**Table 3:** SF-36 scores for 27 patients receiving rituximab induction and maintenance treatment, norm-based and raw scores, at baseline and during follow-up^a^.

**Follow-up (months)**	**Baseline**	**3**	**6**	**10**	**15**	**20**	**24**	**30**	**36** ^**b**^
*Norm-based*, *mean (SD)*	n = 27	n = 26	n = 25	n = 24	n = 24	n = 23	n = 25	n = 18	n = 18
**Physical health summary**	25.6 (6.6)	27.2 (6.8)	32.2 (10.1)	33.1 (12.1)	35.7 (12.3)	37.9 (13.7)	36.4 (12.7)	38.6 (15.7)	38.6 (14.3)
**Mental health summary**	44.6 (10.4)	44.1 (9.6)	47.8 (7.4)	49.2 (6.6)	46.1 (9.3)	49.4 (8.8)	48.7 (8.3)	49.3 (9.1)	47.8 (9.3)
**Physical Function**	33.0 (7.9)	34.7 (9.5)	39.9 (9.4)	40.7 (11.8)	42.8 (11.3)	44.2 (11.5)	43.4 (11.3)	43.5 (12.7)	44.2 (11.3)
**Role Physical**	29.1 (3.4)	29.7 (4.2)	34.0 (9.9)	33.7 (10.6)	35.5 (10.0)	38.3 (12.3)	36.0 (11.2)	37.6 (13.6)	37.6 (12.9)
**Bodily Pain**	32.0 (8.4)	33.0 (8.1)	37.0 (9.0)	39.0 (11.9)	39.6 (12.6)	43.4 (14.0)	41.6 (11.3)	42.7 (14.2)	42.3 (13.3)
**General Health**	31.1 (5.0)	31.4 (5.2)	35.0 (8.5)	36.3 (9.9)	36.6 (10.6)	38.0 (11.7)	37.7 (10.5)	42.5 (14.4)	41.2 (12.9)
**Vitality**	31.6 (5.9)	33.1 (7.0)	40.2 (10.6)	41.6 (11.7)	40.9 (13.0)	44.6 (13.6)	41.8 (12.0)	44.5 (15.8)	41.0 (15.3)
**Social Function**	21.4 (8.7)	22.8 (10.0)	30.5 (12.9)	31.9 (14.5)	31.2 (14.9)	36.5 (14.6)	35.7 (13.4)	38.3 (16.5)	37.7 (16.4)
**Role Emotional**	48.9 (11.1)	48.3 (11.1)	48.8 (11.0)	49.8 (10.0)	48.1 (11.1)	49.6 (10.2)	49.7 (11.1)	49.1 (12.2)	48.0 (13.1)
**Mental Health**	46.9 (10.7)	46.1 (10.2)	50.1 (7.3)	51.4 (7.8)	49.9 (9.6)	51.7 (9.8)	50.8 (9.2)	50.6 (8.6)	50.6 (9.2)
**Follow-up (months)**	**Baseline**	**3**	**6**	**10**	**15**	**20**	**24**	**30**	**36**
*Raw scores*, *mean (SD)*	n = 27	n = 26	n = 25	n = 24	n = 24	n = 23	n = 25	n = 18	n = 18
**Physical Function**	40.6 (19.3)	44.8 (23.2)	57.5 (23.0)	59.6 (29.1)	64.6 (27.8)	68.0 (28.2)	67.1 (27.5)	66.4 (31.3)	68.1 (27.8)
**Role Physical**	4.6 (12.1)	6.7 (15.1)	22.0 (34.9)	20.8 (37.3)	27.1 (35.3)	37.0 (43.3)	31.7 (41.0)	34.7 (47.9)	34.7 (45.5)
**Bodily Pain**	30.0 (20.2)	32.3 (19.4)	42.0 (21.8)	46.9 (28.6)	48.2 (30.4)	57.4 (33.7)	55.0 (34.4)	55.7 (34.3)	54.9 (32.2)
**General Health**	29.8 (10.8)	30.6 (11.2)	38.2 (18.4)	41.1 (21.4)	41.7 (23.0)	44.7 (25.3)	46.3 (24.8)	54.4 (31.1)	51.7 (27.8)
**Vitality**	17.6 (13.0)	20.8 (15.2)	36.2 (23.1)	39.4 (25.5)	37.7 (28.4)	45.9 (29.6)	41.9 (27.8)	45.6 (34.5)	38.1 (33.3)
**Social Function**	20.4 (20.0)	23.6 (22.7)	41.0 (29.4)	44.3 (33.2)	42.7 (34.0)	54.9 (33.2)	54.8 (31.4)	59.0 (37.6)	57.6 (37.4)
**Role Emotional**	79.0 (36.0)	76.9 (36.2)	78.7 (35.9)	81.9 (32.6)	72.2 (38.9)	81.1 (33.1)	84.6 (34.3)	79.6 (39.8)	81.5 (38.3)
**Mental Health**	70.8 (18.5)	69.5 (17.7)	76.5 (12.6)	78.7 (13.4)	76.0 (16.5)	79.1 (16.9)	78.2 (15.8)	77.3 (14.8)	76.9 (16.3)

^a^:SF-36 (Norwegian ver. 1.2) data during follow-up, for 27 patients receiving rituximab induction and maintenance treatment, including 17 patients with, and 10 without, clinically significant response. SF-36 scores (mean, SD) for Physical health summary score, Mental health summary score, and both norm-based scores (with population mean 50, according to US 1998) and raw scores (scale 0–100) for the subdimensions Physical function, Role physical, Bodily pain, General health, Vitality, Social function, Role emotional, and Mental health, are shown. One pilot patient did not fill in SF-36 forms. Including baseline until 24 months follow-up, 15 out of 189 forms were missing (7.9%). Out of 17 patients with clinically significant responses, one withdrew from study after 24 months due to a diagnosis of T2N0 breast cancer, and two patients withdrew from follow-up after 25 and 32 months, respectively. Out of 10 patients with no clinically significant response, one patient withdrew from study after 12 months, and four patients after 24–26 months follow-up. Thus, the mean SF-36 scores at 30 and 36 months will be influenced by fewer non-responders included in the analyses.

^b^: in two patients, the last SF-36 forms were completed at 33 and 35 months, respectively.

**Table 4:** SF-36 raw scores (scale 0–100) in 17 patients with clinically significant responses, and in 10 patients with no significant response, at baseline and during follow-up^a^.

**Follow-up (months)**	**Baseline**	**3**	**6**	**10**	**15**	**20**	**24**	**30**	**36** ^**b**^
***Responders*, *Raw scores***	n = 17	n = 16	n = 16	n = 15	n = 15	n = 15	n = 17	n = 13	n = 14
** Physical Function,** mean (SD)	42.9 (20.7)	46.3 (22.6)	65.3 (22.5)	69.7 (29.1)	78.7 (20.0)	83.3 (14.8)	80.3 (18.4)	72.7 (30.5)	74.2 (24.9)
** Bodily pain**	32.2 (21.4)	33.0 (22.2)	46.9 (23.6)	55.7 (31.8)	58.3 (33.7)	73.3 (28.7)	64.9 (28.9)	61.8 (36.7)	65.0 (33.8)
** General health**	31.8 (12.1)	32.8 (13.0)	45.0 (19.5)	50.1 (22.1)	52.1 (23.2)	56.9 (22.8)	57.9 (22.7)	65.8 (28.6)	63.6 (27.5)
** Vitality**	17.7 (13.4)	21.0 (14.9)	47.5 (20.2)	52.0 (22.8)	50.7 (27.5)	61.3 (23.4)	56.2 (23.0)	56.2 (33.6)	49.6 (35.4)
** Social function**	18.4 (19.8)	22.7 (24.7)	53.1 (27..2)	55.8 (34.7)	60.0 (29.2)	70.8 (25.3)	67.6 (26.5)	68.3 (35.6)	68.8 (36.9)
** Mental health**	68.7 (16.7)	65.5 (16.5)	77.3 (11.3)	80.8 (11.4)	78.9 (9.7)	85.3 (8.5)	81.4 (12.4)	80.6 (11.8)	79.4 (14.7)
**“SF-36mean5”** ^**c**^	28.8 (9.2)				60.1 (22.5)		71.0 (20.8)		62.3 (30.7)
**Follow-up (months)**	**Baseline**	**3**	**6**	**10**	**15**	**20**	**24**	**30**	**36**
***Non-responders*, *Raw scores***	n = 10	n = 10	n = 9	n = 9	n = 9	n = 8	n = 8		
** Physical Function,** mean (SD)	36.5 (16.8)	42.5 (25.2)	43.7 (17.4)	42.8 (21.0)	41.1 (23.0)	39.4 (24.7)	43.2 (26)		
** Bodily Pain**	26.1 (18.6)	31.2 (15.0)	33.0 (14.9)	32.1 (13.7)	33.3 (13.6)	27.5 (19.1)	36.4 (15.4)		
** General Health**	26.5 (7.4)	27.0 (6.3)	25.5 (6.9)	26.1 (7.4)	24.5 (6.0)	21.9 (8.0)	24.4 (8.1)		
** Vitality**	17.5 (13.0)	20.5 (16.6)	16.1 (11.1)	18.3 (12.5)	17.5 (12.3)	16.9 (13.1)	15.0 (10.3)		
** Social Function**	23.8 (20.8)	25.0 (20.4)	23.8 (23.2)	25.0 (19.8)	13.9 (18.2)	25.0 (25.0)	30.6 (25.9)		
** Mental Health**	74.4 (21.5)	76.0 (18.5)	76.4 (14.9)	75.1 (16.2)	71.1 (23.9)	67.5 (22.7)	72.0 (20.3)		
**“SF-36mean5”**	26.1 (10.2)				27.4 (11.0)		30.4 (12.5)		

^a^:SF-36 (Norwegian ver. 1.2) for the subdimensions Physical function, Bodily pain, General health, Vitality, Social function, Mental health, and mean of five subdimentions (“SF-36mean5”), with raw scores (scale 0–100), from 27 patients receiving rituximab induction and maintenance treatment, including 17 patients with clinically significant responses (major and moderate) and 10 patients with no significant response (marginal or no response) during follow-up. One pilot patient did not fill in SF-36 forms. Including follow-up from baseline until 24 months, 15 out of 189 forms were missing (7.9%). Out of 17 patients with clinically significant responses, one patient withdrew due to diagnosis of a T2N0 breast cancer at 24 months follow-up, and two moderate responders withdrew from follow-up after 25 and 32 months, respectively. Out of 10 patients with no clinically significant response, one patient withdrew from study after 12 months, and four patients after 24–26 months follow-up.

^b^: in two patients the last SF-36 forms were completed at 33 and 35 months, respectively.

^c^: “SF-36mean5” denotes mean value of raw scores for the five SF-36 subdimensions Physical function, Bodily pain, Vitality, General health, and Social function.

As expected, the SF-36 scores were low at baseline reflecting the high symptom burden (Table 3, Table 4, Fig 5, S7 Fig). There were no significant differences between baseline SF-36 raw scores for those later achieving a major response, a moderate response, or no response (Fig 5B, 5D, 5F, 5H and 5J).

In the non-responder group, only slight variations in SF-36 raw scores were seen through follow-up (Fig 5, Table 4) with no significant differences from baseline. Interestingly, among the group of major responders, i.e. half of those receiving rituximab maintenance treatment, the increases in SF-36 raw scores from baseline to the “peaks” at approximately 20–24 months were substantial (Fig 5B, 5D, 5F and 5H). Among clinically significant responders (major and moderate), the absolute increases in SF-36 raw scores, from baseline to 20-months follow-up, were highest for the subdimensions Vitality 43.6 (from 17.7 to 61.3), and Social function 52.4 (from 18.4 to 70.8). The corresponding absolute increases were for Physical function 40.4 (from 42.9 to 83.3), for Bodily pain 41.1 (from 32.2 to 73.3), while for Mental health 16.6 (from 68.7 to 85.3) (Table 4). Generally, no differences from baseline were seen at the 3-months follow-up, while the improvements were then seen from 6-months and reaching maximum at 20-24-30 months follow-up, and then a decline in some SF-36 raw scores at end of follow-up.

After 15–20 months follow-up, we had available Sensewear electronic armbands that continuously measured physical activity in the home setting. No data from baseline before intervention were available. The analyses were not preplanned, and were performed only in some patients (mainly in responders). They were performed in order to gain experience with the armbands for design of the protocol for the now ongoing randomized phase III-study. However, 12 out of 14 major responders in this study measured physical activity for 4–6 consecutive days in the time interval 15–20 months follow-up, with a mean value for “mean number of steps per 24h” 9829 (range 5794–18177), and a mean value for “maximum number of steps per 24h” 14623 (range 9310–23407).

**Fig 6:**
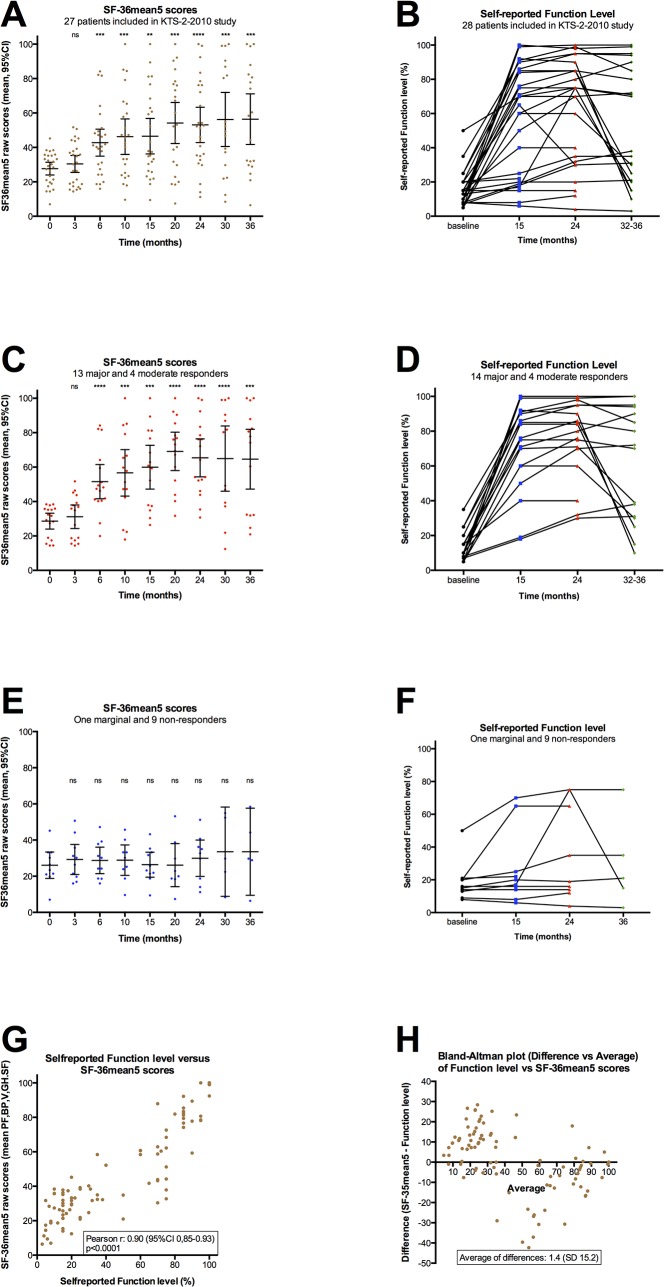
Function level and “SF-36mean5”. Mean values of SF-36 raw scores for the five subdimensions Physical function (PF), Bodily pain (BP), Vitality (V), Social function (SF) and General health (GH) are shown (denoted “SF-36mean5”, scale 0–100), at baseline and at 3, 6, 10, 15, 20, 24, 30 and 36 months follow-up. SF-36mean5 scores for each time point during follow-up were compared to baseline scores. P-values were calculated using Repeated Measures One-way ANOVA, with Dunnett’s multiple comparison adjustments, and are indicated at the top of each panel. ns: not significant; **: p<0.01; ***: p<0.001; ****: p<0.0001. To be able to analyze for differences at each time point relative to baseline, a missing value at a time point for a patient was replaced with value interpolated between the previous and next values during follow-up for that patient (but not replaced in the plot). Panel A shows “SF-36mean5” (raw) scores for 27 patients in this study. One pilot patient did not fill in SF-36 forms. In addition 22/243 (9.1%) data were missing, including for patients out of study before end of follow-up, as explained in M&M. Panel C shows “SF-36mean5” scores for 17 patients with clinically significant response (data from pilot 2, and in addition 10/153 (6.5%) data were missing). Panel E shows “SF-36mean5” scores for 10 patients with no clinically significant response during follow-up (12/90 (13.3%) data were missing). Panels B, D and F show self-reported Function levels (according to a form with examples, see S3 Fig), at baseline and at 15, 24 and 36 months. In panel B for 28 patients included in the KTS-2-2010 study, in panel D for 18 patients with clinically significant responses, and in panel F for 10 patients without clinically significant responses. In panels B and D, two moderate responders registered their Function level at 32 and 33 months (instead of 36 months), respectively. Panel G shows a correlation plot between “SF-36mean5” raw scores and self-reported Function levels (both with scale 0–100), with pooled data from baseline and at 15, 24 and 36 months follow-up. Panel H shows a Bland-Altman plot for difference (“SF-36mean5”—Function level) versus average.

These activity data are not valid for formal response characterization, but the counted number of steps per day for major responders corresponded with the level found in the normal population, and thus support the SF-36 data also showing that major responders in the time interval 15–30 months follow-up report SF-36 subdimensions at the mean level of the general population (Table 1, Table 4, Fig 5, Fig 6, S7 Fig).

### Self-reported Function level

For the 28 patients receiving rituximab induction and maintenance, the self-reported Function levels at baseline are shown in Table 2. Function levels, according to a form with examples (S3 Fig) in which 100% denotes a completely healthy state, reported at 15, 24 and 36 months are shown in Fig 6, with lines corresponding to each patient. Fig 6, panel B shows Function levels for all included 28 patients, panel D shows separate data for the 18 patients with clinically significant responses, and panel F shows data for non-responders.

To assess which SF-36 subdimensions best described the baseline status and clinical course during follow-up, we looked at correlations between SF-36 subdimensions (raw scores, scale 0–100) and self-reported Function level (scale 0–100), at baseline and at 15, 24, and 36 months. The SF-36 subdimensions Role-emotional and Role-physical have previously been reported as inaccurate [15], and these also impact on the SF-36 summary scores. The best correlation (data not shown) was found between self-reported Function level and a mean of SF-36 raw scores for Physical function, Bodily pain, Vitality, Social function and General health (denoted “SF-36mean5”). Fig 6G shows the correlation plot from the pooled data at baseline, 15, 24 and 36 months, with a mean difference (“SF-36mean5”–Function level) of only 1.4 (SD 15.2). A plot of difference versus average showed that the “SF-36mean5” tended to report higher values in the low range, while Function level reported higher values in the mid-range, while the overall mean agreement was good (Fig 6H). The “SF-36mean5x scores during follow-up for 27 patients are shown in Fig 6A. P-values for comparison of each time point to baseline are indicated (multiple comparison adjusted). Similar plots for 17 patients with major and moderate response (Fig 6C), and for 10 patients with no clinically significant response (Fig 6E) are shown.

There were no significant differences between “SF-36mean5” data at baseline and at 3 months follow-up. Thereafter, i.e. at 6, 10, 15, 20, 24 and 36 months, there were highly significant differences in “SF-36mean5” scores as compared to baseline, for all patients, and for major and moderate responders (Fig 6A, and 6C). In the non-responder group, there were no significant differences in “SF-36mean5” scores as compared to baseline, at any time point during follow-up (Fig 6E).

### Adverse effects

Two patients experienced an episode of late-onset neutropenia (LON). One pilot patient (major responder) had LON after 34 months follow-up (Table 1, S4 Fig panel A), and one major responder after approximately 22 months (Table 1, S4 Fig panel E). In both patients the registered LON lasted for five days before recovery of neutrophil counts, and was uncomplicated.

Two patients had several upper airways infections. In one major responder (S4 Fig panel C) three repeated antibiotic courses were given for sinusitis. She was then given normal human immunoglobulins (20 g) twice, after which the symptoms resolved and she received in total five rituximab infusions. One non-responder (S6 Fig panel W) had several courses of oral antibiotics due to respiratory tract infections, also seemingly with therapeutic benefit from two infusions of normal human immunoglobulins (20 g). This patient, however, also had a tendency for repeated upper respiratory tract infections before entering the study. One patient had an uncomplicated episode of upper urinary tract infection.

Eight out of 28 patients (29%) receiving rituximab induction and maintenance infusions experienced varying degrees of ME/CFS symptom worsening the first days or weeks after a new infusion. This phenomenon was pronounced after most of the infusions in two patients (Table 1, S4 Fig panels K,L), but seen only after one or some of the rituximab infusions in six patients (Table 1, S4 Fig panels B,F,G,I, S5 Fig panel O,Q). Transient symptom worsening was also seen after one ofatumumab infusion in one patient (S4 Fig, panel J).

Two patients experienced an allergic reaction during rituximab infusions, as described above (S4 Fig, panel J). One patient (major responder after both rituximab and ofatumumab infusions) was diagnosed with a breast cancer (pT2N0, ER+) at 24 months follow-up, probably not related to rituximab infusions (Table 1, S4 Fig panel J). One non-responder (Table 1, S6 Fig panel W) had idiopathic thrombocytopenic purpura at end of follow-up (at 36 months). One moderate responder (Table 1, S5 Fig panel R) had an episode of choledocholithiasis at 33 months follow-up.

### B-lymphocytes

**Fig 7:**
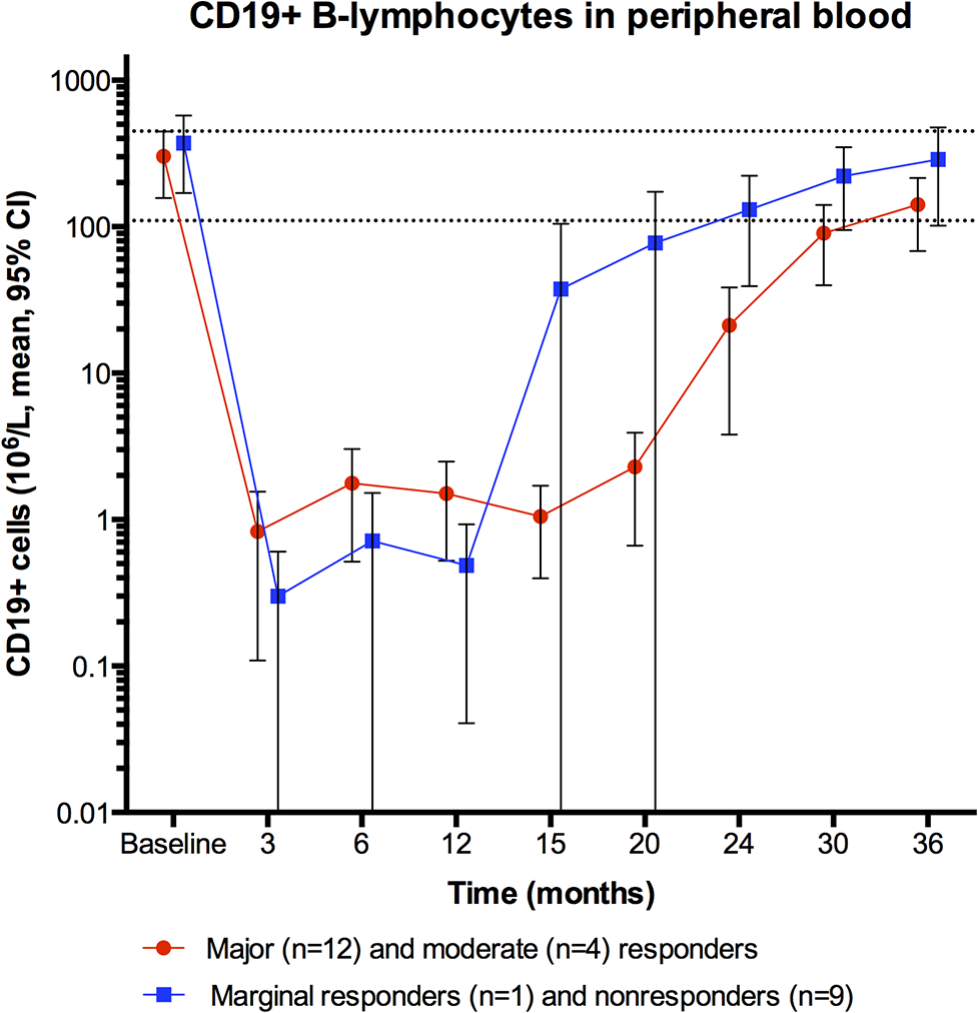
CD19+ B-lymphocytes in peripheral blood during follow-up. B-lymphocyte numbers from immunophenotyping of peripheral blood during follow-up are shown, at baseline and 3, 6, 12, 15, 20, 24, 30 and 36 months follow-up. The red dots represent mean value at each time point, for patients with either major or moderate clinical response (n = 16). The blue squares denote the mean values for patients with no significant response (n = 10). The value zero for B-lymphocytes in peripheral blood was substituted by 0.1 (to enable plotting on the log scale). B-lymphocyte counts x 10^6^/L (normal range 110–449). The error bars denote mean ± 95% CI. B-cell data during follow-up were not available for the two pilot patients (both major responders). According to an amendment, seven patients received further rituximab infusions in addition to the six infusions stated in the initial protocol. Two major responders received five and four rituximab additional rituximab infusions, respectively. Four moderate responders received five, four, three and three additional rituximab infusions. One non-responder received two additional rituximab infusions.

B-lymphocyte counts in peripheral blood during follow-up, assessed from flowcytometry for CD19+ cells, are shown in Fig 7. B-cell data from patients with clinically significant responses (n = 18) and non-responders (n = 10) are shown. The difference in B-cell regeneration between responders and non-responders may not be interpreted, because the patient groups have received different numbers of rituximab-infusions, as described above. However, all patients have recovered B-cell numbers in peripheral blood after end of follow-up.

## Discussion

In the present open-label phase II study (no randomization) with rituximab induction and maintenance infusions and follow-up for 36 months, prolonged B-lymphocyte depletion was associated with distinctly prolonged duration of clinical responses of ME/CFS symptoms, as compared with data from the previous randomized phase II study in which patients only received two rituximab infusions two weeks apart [7].

According to the predefined criteria, a clinical response was detected in 62% (18 out of 29 included with intention to treat). Clinically significant responses, determined post-hoc, were seen in 64%, i.e. in 18 out of 28 patients receiving maintenance treatment. Thus, the response is similar to the 67% detected in the previous randomized, placebo-controlled trial [7].

The interpretation of clinically meaningful responses was supported by SF-36 data. At baseline, ME/CFS patients in this study reported low SF-36 scores, compatible with a high level of symptom burden, in accordance with other studies [4]. The lowest baseline SF-36 scores were detected for the subdimensions Vitality and Social function. For the subdimension Mental health, the baseline values were at the population mean for moderate responders and non-responders, while a slightly decreased value was detected in those later achieving a major response. Interestingly, no differences from baseline were detected for any of the SF-36 subdimensions at 3 months follow-up. However, increases in SF-36 scores were thereafter seen for the subdimensions Physical function, Bodily pain, Vitality, Social function and General health, which all reached maximum scores at approximately 20–30 months follow-up. For the group of major responders, which comprised half of all included patients, the SF-36 norm-based values were at the population mean during this time interval, supporting the patients’ subjective interpretation of substantial improvement in ME/CFS symptoms. The absolute increases in raw scores of SF-36 subdimensions also clearly demonstrate the differences in symptom improvements during follow-up, between the 2/3 of patients with clinically significant responses, versus the 1/3 with no benefit from the intervention. While there were no differences in SF-36 raw scores through follow-up in the non-responders, the group with major responses showed substantial absolute increases in SF-36 raw scores, with absolute differences between 20 months and baseline of 43 (Physical function), 50 (Bodily pain), 53 (Vitality) and 60 (Social function).

Also, the self-reported “Function level” registered by the patients at baseline and at 15, 24 and 36 months, indicated that self-reported clinical responses correlated with an increase in functioning with impact on daily life. The calculated “mean of five SF-36 subdimensions” (raw scores) for Physical function, Bodily pain, Vitality, Social function and General health (denoted “SF-36mean5”) showed the best correlation with self-reported “Function levels” in pooled data from baseline and from 15, 24 and 36 months follow-up. Because the Physical Health Summary score and Mental Health Summary score are influenced also by the SF-36 subdimensions Role-physical and Role-emotional, which have previously been reported as inaccurate in patients with severe functional impairment syndromes [15], it is not surprising that an expression taking into account these five subdimensions better described the patients’ own experience of daily level of functioning.

The response rate in this study may be influenced by inclusion of patients who participated in the previous randomized and placebo-controlled study [7]. However, among nine patients in the placebo group in the previous KTS-1-2008 study, all of which had shown no signs of clinical response during 12 months follow-up when given placebo, six of these achieved a clinically significant response during the first 12 months when given rituximab maintenance treatment in the present study. In addition, one patient reached a clinically significant response at later than 12 months follow-up. These data support an influence of B-cell depletion on the mechanism for ME/CFS symptom maintenance.

Ten patients previously given rituximab, including three with no clinically significant responses in KTS-1-2008, participated in the present study to investigate whether prolonged B-cell depletion could benefit these. Out of three non-responders in KTS-1-2008, one reached a clear clinical response after rituximab maintenance in the present study.

Among seven patients given prolonged B-cell depletion due to slow and gradual improvement of symptoms after 12 months (according to an approved amendment) most remained as moderate responders throughout the study, suggesting that prolonged rituximab maintenance phase may not give major additional benefit to this group.

Within the three years follow-up period, the mean duration of responses was 97 weeks in the group of major and moderate responders. In addition, out of 18 patients with clinically significant responses, 11 were still in sustained stable responses at the end of follow-up. Three had experienced partial relapse and four almost full relapse of ME/CFS symptoms. Thus, maintenance rituximab treatment with a prolonged period of B-cell depletion seems to be associated with significantly prolonged response durations compared to two infusions rituximab two weeks apart. In the previous study [7], the mean response duration was 25 weeks within 12 months follow-up, and with response duration beyond the 12 months study period in only four out of ten responders.

In the present study, B-lymphocyte counts in peripheral blood were recovered in all patients after end of follow-up, and possible mechanisms for sustained clinical responses are not obvious. Pilot immunophenotyping analyses of B-cell subsets (naïve versus memory versus transitional versus plasmablasts) after B-cell regeneration (i.e. at 36-40-44 months follow-up) are in progress, aiming to investigate possible differences between sustained responders, patients with partial or complete relapse, and non-responders.

The relationships between rituximab treatment, B-cell depletion, B-cell subsets and clinical responses have been studied in rheumatoid arthritis (RA) [16]. An increased fraction of memory cells was detected in those with early relapses [17]. In B-cell depletion naïve RA-patients, low levels of CD27+ memory B-cells may predict later response to rituximab treatment [18]. Rituximab targets short-lived autoreactive plasma cells more consistently than the more long-lived protective plasma cells [19,20]. In systemic lupus (SLE), B-cell clones producing autoantibodies had a more rapid turnover than B-cells producing protective antibodies [21], making autoreactive B-cells more vulnerable to rituximab treatment.

For major responders, a mean time lag of 23 weeks (median 19 weeks, range 8–66) between initial and rapid B-cell depletion in peripheral blood and start of clinical responses is an argument in favor of a possible autoimmune pathogenesis in a subgroup of ME/CFS patients. The patterns of responses and relapses seen in this study are in accordance with response patterns reported for some established autoimmune diseases, such as Wegener’s granulomatosis [22]. The lag time before clinical improvement may be compatible with a mechanism involving reduction of long-lived autoantibodies, or some other slow alteration in immune function governed by B-cells.

An observation that may support a variant of an autoimmune mechanism underlying the entity is a moderate but highly significant increase in risk of B-cell lymphomas in elderly CFS patients, indicating a chronically activated B-cell system [23]. In this population-based case-control study among almost 1.2 million cancer cases aged more than 65 years, and 100.000 elderly controls without cancer, with a prevalence of CFS 0.5% in both groups, a modest but highly significant association between CFS and non-Hodgkin lymphoma was reported. There were no significant associations to other cancer types when adjusting for multiple comparisons. Of lymphoma subtypes, a significant association to the most common aggressive lymphoma (diffuse large B-cell lymphoma) was reported. Interestingly, there was a highly significant association between CFS and marginal-zone lymphomas [23]. This low-grade B-cell lymphoma type often arise in extra-nodal tissues, in which chronic stimulation by an antigen is thought to play an essential role in lymphomagenesis either from chronic infections or from autoimmunity [24].

Among patients included in the present study, 41% had one or more first-degree relatives with an autoimmune disease (AD). The prevalence of known AD in the general population was estimated to 3.2% in a study from US [25] and to at least 5% in a study from Denmark [26]. Taking into account that each individual may have on average 4–6 first-degree relatives, and also that several AD to some extent tend to cluster in families, the reported 41% in this study is probably higher than expected and therefore may indicate a genetic predisposition for AD.

Interestingly, recent studies have indicated a possible autoimmune basis for Postural Tachycardia Syndrome (POTS) with autoantibodies to autonomic receptors [27]. Studies have also suggested that in subsets of Chronic Regional Pain Syndrome (CRPS) patients, associations to partly agonistic autoantibodies to β2-adrenergic receptors and to muscarinic-2 receptors were reported [28]. CRPS has been shown to improve after intravenous immunoglobulin therapy, and has been proposed as a prototype of a novel kind of autoimmunity with a possible two-hit process involving pre-existing autoantibodies that may become pathogenic after a triggering event such as trauma or infection [29]. It is worth noting that POTS is detected in approximately 15% of ME/CFS patients [30], and both POTS and CRPS are seen primarily in young women and have features that partly overlap with ME/CFS such as fatigue, brain fog, and central sensitization.

Several studies have provided data suggesting autoimmunity as a possible mechanism in a subgroup of ME/CFS. Altered B-cell subtypes compared to healthy controls have been detected [31], also reduced NK-cell activity and elevated T-regulatory cells [32]. Infection-induced autoimmunity was suggested as a possible disease mechanism in a recent study reporting IgM to human and microbial heat shock protein 60 (HSP60) in a high frequency of ME/CFS patients as compared to healthy [33]. Increased IgM-mediated autoimmune responses to oxidative specific epitopes were recently proposed to be involved in the pathophysiology of ME/CFS [34]. A recent study investigated cytokine patterns in peripheral blood from ME/CFS patients [35], showing distinct abnormalities of both pro- and anti-inflammatory cytokines early in the course of ME/CFS, which were not present after the first three years. The same research group also showed a disturbed cytokine pattern in cerebrospinal fluid of ME/CFS patients, compared to healthy controls and multiple sclerosis patients. The findings were consistent with an immune activation and a shift towards a Th-2 pattern, which may be associated with autoimmunity [36].

However, other underlying mechanisms than autoimmunity may explain the observed clinical effects of B-cell depletion on ME/CFS symptom maintenance. Rituximab influences other aspects of immune function than the pronounced effect on CD20 positive B-cells. These include rituximab-induced T-cell inactivation, T-cell polarization to a suppressive phenotype, elimination of B-lymphocytes as antigen-presenting cells, and depletion of a CD20dim T-cell subset [37]. B-cell depletion impairs adaptive and autoreactive CD4-positive cells in mice, as one mechanism for observed clinical benefit of rituximab in presumed T-cell mediated autoimmune diseases [38]. Whatever the mechanism behind the rituximab effect, the patient described in our study with a response but allergic reaction to rituximab, and later treated with the humanized anti-CD20 antibody ofatumumab again achieving a clinical response, indicate B-cell depletion as the factor responsible for response and not some other, unknown effect of the antibody.

There were no severe or unexpected side effects from rituximab maintenance treatment in this study. Two episodes of uncomplicated late-onset neutropenia (LON) were observed, both lasting for five days before recovery of neutrophil counts. The incidence of LON varies with the clinical setting, but seems to be comparable in rheumatologic and hematologic diseases [39]. LON occurred in 9% of B-cell lymphoma patients treated with rituximab at a median 121 days (range 49–474 days) from end of rituximab treatment [40].

We observed few infections, and no cases of septicemia or life-threatening infections. Two patients had several upper airways infections demanding antibiotics, both seemed to benefit from two infusions of intravenous normal human immunoglobulins. In the present study, serum immunoglobulins were measured at all visits. Generally, there were slight but significant decreases in levels of IgG, IgA or IgM during follow-up (manuscript in preparation).

Two patients experienced an allergic reaction to rituximab-infusions. In the majority of patients no infusion-related symptoms or subsequent ME/CFS worsening could be noted. In eight patients, symptom flares were observed after one or more rituximab infusions. This is infrequent in lymphoma patients receiving rituximab. Such transient worsening also occurred when a patient was B-cell depleted and had a sustained major response, and usually lasted for days to a few weeks. However, we have observed one patient included in another study (KTS-3-2010, for patients with severe or very severe ME/CFS) who experienced such transient symptom worsening lasting for almost three months. Based on pilot observations, a slow infusion rate of rituximab seems to reduce this problem, and in the presently ongoing randomized phase III study (“RituxME”) all intravenous infusions are given over approximately four to five hours infusion time. The symptom worsening seems similar to that described after infusion of normal human immunoglobulins as a therapeutic option in adolescent ME/CFS [41] and may indicate an unspecific immunoglobulin effect in these patients.

Rituximab maintenance treatment is generally considered safe. However, even if severe adverse effects are rare, they may occur and include defects in immune reconstitution, and reactivation of chronic viral infections such as hepatitis [42]. B-cell depletion using rituximab is effective and safe in Sjøgren’s syndrome [43], and also in RA patients although repeated infusions over time was associated with hypogammaglobulinemia [44]. In a review article, rituximab treatment for two years was considered safe in multiple sclerosis (MS) with no severe toxicity. Substantial benefit was reported on relapsing-remitting MS, but with marginal effect on primary progressive MS [45]. In a study of patients treated with rituximab for rheumatoid arthritis in several centers in the US, Europe and Australia, describing development of the often lethal progressive multifocal encephalopathy (PML) caused by JC-virus reactivation in the brain, four cases were detected among an estimated population at risk of 129.000, suggesting an increased risk of about one case per 25.000 individuals [46]. However, the majority of these patients had also received additional immunosuppressive drugs.

A major limitation of the present study is the open-label design and lack of a placebo group for comparison. The inclusion of patients from both the placebo and rituximab-groups from the previous KTS-1-2008 randomized phase-II study [7] (in total 18 out of 29 patients) may preclude the estimate of the response rate to rituximab maintenance treatment. The previously published KTS-1-2008 study [7] had several limitations, being the first to investigate the treatment principle of B-cell depletion in ME/CFS, with inadequate predefinition of all endpoints and also a negative primary endpoint (i.e. no difference between groups at 3-months follow-up). Also, any selection bias in our two studies is unknown. The (probably) high frequency of autoimmune diseases among first- and second-degree relatives of ME/CFS patients in our studies could be a sign of unintended selection bias. Alternatively, it might prove to be a characteristic also in a broader population of ME/CFS patients, as detailed family histories are not always taken in studies.

Therefore, to verify or refute the results from the previous randomized phase II and the present open-label phase II study, a new randomized, double-blind, and placebo-controlled phase III study in five centers in Norway, including four university hospitals, has been launched (“RituxME”, NCT02229942). In this study, 152 patients will be randomized 1:1 between rituximab or placebo, two infusions two weeks apart, with maintenance infusions after 3, 6, 9 and 12 months, and with follow-up for 24 months. In addition to self-reported symptom scores recorded every second week and SF-36 questionnaires with 3-month intervals, physical activity will be measured using an armband-computer for seven consecutive days, at baseline and repeated in the interval 17–21 months. Three substudies are conducted aiming to evaluate vascular endothelial function, gastrointestinal function, and performance in cardiopulmonary exercise tests for two consecutive days (only in mild-moderate, not in severe ME/CFS), all assessments performed at baseline and repeated in the interval 17–21 months after start of intervention (i.e. during double-blind design).

We are also conducting an open-label phase II study (KTS-3-2010) aiming to include only patients with either very severe or severe ME/CFS, using the same treatment regimen with rituximab induction and maintenance as described in the study presented here (KTS-2-2010). Patients with very severe ME/CFS have special needs, and both transporting and accommodating them in a busy oncology ward have proved very difficult. Eight patients have been included, and only four with very severe ME/CFS have been given rituximab maintenance treatment in KTS-3-2010. Although the treatment had a slight beneficial effect on two out of four patients with very severe ME/CFS, none of the four will be characterized as responders. B-cell depletion using rituximab for ME/CFS is at present an experimental treatment, and more evidence is needed. We do not encourage the use of rituximab for ME/CFS outside of approved clinical trials, and this is especially important for the group with very severe disease.

In conclusion, we believe that the findings presented herein strengthen our previous observations, demonstrating that use of the monoclonal anti-CD20 antibody rituximab may give clinical benefit in a subgroup of patients with ME/CFS. The results indicate that prolonged B-cell depletion with maintenance rituximab infusions is associated with longer response durations than induction treatment alone, with no major or unexpected toxicity. However, one-third of the study patients still show no significant clinical response to rituximab treatment. The observed patterns of response and relapse with a time lag before clinical improvement, along with a female preponderance, a higher than expected occurrence of autoimmune diseases in near family (in our studies), and published data showing a moderate, but highly significant increased risk of B-cell lymphomas in elderly ME/CFS patients, suggest to us that ME/CFS in a subgroup of patients may be a variant of an autoimmune disease. It may involve antibodies, is often triggered by infections, and probably involves a genetic predisposition. An important scientific issue will be to elucidate the target for the putative pathologic immune response, i.e. to understand the effector system for symptom maintenance and how the immune process disturbs this system, and thus explains the various aspects of clinical presentation in this devastating disease. An understanding of the disease mechanisms may also pave the way for further rational treatment strategies.

## Supporting Information

S1 Protocol Protocol for the KTS-2-2010 study (April 2010), including one amendment (December 2011). (PDF)

S1 TREND Checklist Trend checklist for the KTS-2-2010 study. (PDF)

S1 Fig Form for patient’s self-reported ME/CFS symptoms at baseline. Before intervention, the patients assessed their ME/CFS disease and recorded their symptoms during the preceding three months period, using the scale 1–10 (1: no symptom; 5: moderate symptom; 10: very severe symptom).
(TIF)

S2 Fig Form for patient’s self-reported ME/CFS symptom changes every second week during follow-up. During 36 months follow-up, the patients recorded symptom changes every two weeks, always as compared to baseline. The scale for the follow-up form was 0–6 (0: Major worsening; 1: Moderate worsening; 2: Slight worsening; 3: No change from baseline; 4: Slight improvement; 5: Moderate improvement; 6: Major improvement).
(TIF)

S3 Fig Self-reported Function level, form with examples. The patients assessed their Function level (scale 0–100%) in which 100% denoted completely healthy as before the patient acquired ME/CFS, according to examples in this form. The Function levels were assessed at baseline, and at 15, 24 and 36 months follow-up.
(TIF)

S4 Fig Self-reported symptom scores, Function levels, and SF-36 scores, for 12 patients with major clinical responses during follow-up. Panels A-L show follow-up data for each of 12 patients with major clinical responses during 36 months follow-up. In each panel the lines represent self-reported symptom scores. Every second week, the patients recorded symptom changes, always compared to baseline, in a separate form (S2 Fig). *Fatigue score* (black line) was calculated every second week as the mean of four fatigue-related symptoms (Fatigue, Post-exertional malaise, Need for rest, Daily functioning). *Cognitive score* (red line) was calculated as the mean of three symptoms (Concentration ability, Memory disturbance, Mental tiredness). *Pain score* (green line) was calculated as the mean of the two dominant pain symptoms (if pre-treatment level ≥ 4). The vertical bars (scales 0–100) represent the SF-36 raw scores for Physical function (blue bars), “SF-36mean5” (i.e. mean of raw scores for Physical function, Bodily pain, Vitality, General health, Social function) (purple bars), and self-reported Function level according to a form with examples (S3 Fig) (orange bars), at baseline, and at 15, 24 and 36 months follow-up. “R” in the panels indicates time points for rituximab infusions.
(TIF)

S5 Fig Self-reported symptom scores, Function levels, and SF-36 scores, for six patients (responders) receiving additional rituximab-infusions. Panels M-R show follow-up data for each of for six patients receiving additional rituximab-infusions, according to a study amendment, including two patients with major response, and four patients with moderate response. See legend to S4 Fig.
(TIF)

S6 Fig Self-reported symptom scores, Function levels, and SF-36 scores, for 10 patients with no clinically significant responses. Panels S-Z3 show follow-up data for each of 10 patients with no clinically significant response, including one patient with “marginal” response, and nine patients with no response during follow-up. See legend to S4 Fig.
(TIF)

S7 Fig SF-36 questionnaire, norm-based scores. SF-36 (Norwegian ver. 1.2) forms were recorded at baseline and at 3, 6, 10, 15, 20, 24, 30 and 36 months. Norm-based SF-36 scores (according to US 1998) are shown for 27 patients included in the KTS-2-2010 study, for the subdimensions Physical function (panel A), Bodily pain (panel C), Vitality (panel E), Social function (panel G) and Mental health (panel I). In panels B, D, F, H and J the corresponding SF-36 norm-based scores are shown separately for 13 major responders, four moderate responders, and 10 patients with no clinical significant response (one marginal responders and nine non-responders). The horizontal line in each panel denotes the approximate population means for SF-36 norm-based scores (50). One pilot patient (major responder, withdrew after 32 months) did not fill in SF-36 forms. One included patient did not receive induction rituximab infusions due to an allergic reaction to the first infusion, and did not fill in SF-36 forms. One major responder was diagnosed with a T2N0 breast cancer after 24 months follow-up and withdrew from study to start cancer treatment. Out of four moderate responders, one withdrew from the study after 25 months, and one after 32 months. Out of 10 patients with no clinically significant response one withdrew from study after 12 months, and four patients after 24–26 months follow-up.
(TIF)
