# Supplementary material for: B-Lymphocyte Depletion in Myalgic Encephalopathy/ Chronic Fatigue Syndrome. An Open-Label Phase II Study with Rituximab Maintenance Treatment
Source: PLoS One. 2015 Jul 1;10(7):e0129898. doi: 10.1371/journal.pone.0129898 (PMC4488509; doi:10.1371/journal.pone.0129898)
Supplement: S1 Protocol — (PDF) [file pone.0129898.s001.pdf]

PROTOCOL: KTS-2-2010, version 1, dated 29.04.2010
# - INCLUDING PROTOCOL AMENDMENT dated 08.12.2011

B-LYMPHOCYTE DEPLETION USING THE ANTI-CD20 ANTIBODY RITUXIMAB IN CHRONIC FATIGUE SYNDROME (KTS-2-2010)

-AN OPEN PHASE II STUDY WITH RITUXIMAB INDUCTION AND MAINTENANCE TREATMENT

EudraCT: 2010-020481-17

STUDY CENTRE
The study is managed from the Dept. of Oncology at Haukeland University Hospital (HUS).

INVESTIGATORS
- Prof. Olav Mella, MD, Head of the Dept. of Oncology, HUS (Principal Investigator).
- Senior Consultant Øystein Fluge, MD, Dept. of Oncology, HUS (Study Coordinator).
- Senior Consultant Annette Storstein, MD, Dept. of Neurology, HUS
- Prof. Olav Dahl, MD, Dept. of Oncology, HUS

Responsible for additional substudy with a functional cerebral MRI:
- Senior Consultant Gesche Neckelmann, Dept. of Radiology, HUS
- Prof. Kenneth Hugdahl, Dept. of Biological and Medical Psychology, University of Bergen

BACKGROUND
<u>Please refer to separate project description:</u>
"B-cell depletion using the monoclonal anti-CD20 antibody Rituximab in Chronic Fatigue Syndrome".

Chronic Fatigue Syndrome (CFS) is a condition with unknown etiology, frequently involving considerable symptoms and lasting impaired function or disability. Dysregulation of the immune system, often triggered by exogenous stimuli such as a viral infection, is a possible pathogenetic factor in Chronic Fatigue Syndrome (Myalgic Encephalopathy (ME)). Data from the literature are inconclusive, but

indicate a dysregulation in T and NK cell systems as well as in B cells. Our research group at the Dept. of Oncology, HUS, were the first to publish a paper on the use of B-cell depletion as therapeutic intervention in CFS [1]. The considerable symptom change in three pilot patients indicates that B-lymphocytes play an important part in symptom maintenance in this chronic, debilitating illness. The study also points to probable mechanisms behind the development of CFS.

As a consequence of the pilot study, we are currently, in cooperation with the Dept. of Neurology (HUS), conducting a double-blind, randomized, placebo controlled phase II study on B-lymphocyte depletion using the monoclonal antibody rituximab (Mabthera ®) in patients with CFS (KTS-1-2008, EudraCT 2007-007973-22). The first patient was included in June 2008, and the inclusion phase has been completed (the last patient included in June 2009). The patients are currently being observed for up to 12 months after intervention. The study has now been unblinded to the investigators (mid-February 2010, after the last patient attended the 8 month control visit), while patients remain blinded to treatment allocation until 12 months after intervention.

Interim analysis results confirm that Rituximab is associated with significant clinical response in part of the CFS patient group. Based on clinical assessment, the chief effect parameter is the ”overall response” (i.e. response occurring at any time during follow-up). The results (which must for now be treated with confidence) show that 8 out of 15 patients in the rituximab group have experienced a significant response (“overall response”), while 1 out of 15 patients allocated to the placebo group satisfy the criteria for significant response (p=0.017, Fischer's exact test). Moreover, two patients in the rituximab group and one patient in the placebo group have experienced a “moderate” response (in total 10/15 vs 2/15, p=0.009).

There is a statistically significant difference in response rate at 7 and 8 months follow-up after intervention, based on the patients' self-reported symptom changes recorded every two weeks. The doctor's symptom registration forms also show a significant difference between the groups at 6 and 8 months control visits (secondary endpoints).

The study does not meet its primary endpoint, which is defined the effect on the patients' symptoms after 3 months follow up. There is no difference between the Rituximab and placebo groups with regards to self-reported symptom change or doctor's registration of symptom changes at 3 months compared to baseline. The primary endpoint was defined at a time where we only had knowledge derived from the treatment of two pilot patients. It is worth noting that «improvements» lasting only a few weeks during the first 4-5 months after the intervention also occurred in the placebo group. However, these improvements do not satisfy the response criteria due to the short duration, but affect the statistical analyses until 5 months follow-up.

In conclusion, we find a significant difference in favor of the Rituximab group for ”overall response” and at the secondary endpoints: symptom change 6 and 8 months after intervention.

The patients experiencing significant response noticed a change starting from between 2 and 6 months after intervention. Two patients in the Rituximab group have experienced a major response from approx. 4 months after the intervention, both of whom show no sign of relapse after more than 80 weeks' follow-up. The patients have experienced different response duration (e.g. 2 - 6 months, 3 - 10 months, 3 - 11 months, 6 - 8,5 months, 5-7 months) before relapse.

In this study we have not observed any serious toxicity, and there were no hospitalizations or serious infections during follow-up. Two patients, both in the rituximab group, had a transient flare-up of pre-existing psoriasis, which could be a side-effect of the rituximab intervention.
The study has provided ample material for research, and biological parameters observed at specific times during follow-up may be correlated to clinical response (or lack of such).

In October 2010 it was claimed that CFS patients were infected with a retrovirus (XMRV) causing their symptoms [2]. The use of B-cell depletion in patients with a persisting viral infection would then be relatively contraindicated. However, three recent articles found no incidence of XMRV in cohorts of CFS patients in England and the Netherlands [3-5]. Our patients in the pilot series and the randomized study (33 in total) have all been tested for XMRV with multiple PCR assays using both DNA and RNA templates, all of which came out negative.

Two of the patients included in the published pilot study [1] have experienced a documented effect of repeated rituximab infusions. Evaluation of these patients indicates that maintenance treatment can prolong and maintain a major clinical response, with considerable benefit to the patients with regards to quality of life, physical function, participation in work or study, social life and family life.

The hypothesis for KTS-2-2010 is that induction treatment with Rituximab (two infusions with two weeks' interval) followed by maintenance infusions as detailed (up to 15 months) will cause a lasting and considerable response in the patients. Furthermore, the study aims to procure biological material (blood samples) before intervention and throughout follow-up, in extension of the existing biobank. We will use the biobank material in further research into the pathogenesis and possible biomarkers of the illness.

OBJECTIVES AND ENDPOINTS

Primary endpoint:
-to investigate the effect on the symptoms of Chronic Fatigue Syndrome, after two infusions of the monoclonal anti-CD20 antibody Rituximab (Mabthera®) (500 mg/m^2, max 1000 mg per infusion) given at two weeks' interval, followed by maintenance infusions of Rituximab (500 mg/m^2, max 1000 mg per infusion) at 3, 6, 10 and 15 months.
The primary endpoint "clinical response" is not predefined to any specific time point or interval during follow up, but the response must be recorded as significant on the patient's self-report form (mean fatiguescore $\geq$ 4,5 for at least 6 consecutive weeks, which must include the registration of mean fatiguescore > 5 at some point). Single response periods and the sum of such periods will be recorded.

AMENDMENT dated 08.12.11: *For patients who have taken part in the KTS-2-2010 or KTS-3-2010 studies and experienced a slow and gradual improvement, but who could not be classed as major clinical responders according to the predefined criteria after 12 months follow-up (including five Rituximab infusions), we wish to*

intensify the Rituximab treatment during the following year. The aim is to investigate whether these patients can still achieve a clinically major response. We wish to administer up to 6 additional Rituximab infusions (500 mg/m^2, max 1000 mg) with at least two months intervals, during the following 12 months.

Secondary endpoints:
-to investigate the effect on the symptoms of Chronic Fatigue Syndrome, at evaluations 3, 6, 10, 15, 20, 24, 30 and 36 months after start intervention; after two infusions of the monoclonal anti-CD20 antibody Rituximab (Mabthera®) (500 mg/m^2, max 1000 mg per infusion) given at two weeks' interval, followed by maintenance infusions of Rituximab (500 mg/m^2, max 1000 mg per infusion) at 3, 6, 10 and 15 months.
-to investigate the duration of the longest consecutive clinical response period (mean fatiguescore ≥ 4,5, including mean fatigue score >5).
-to investigate the fraction of included patients still in response at the end of study (36 months after intervention).
The therapeutic effect (for primary and secondary endpoints) is recorded using patient self-report forms and clinical assessment.

-to evaluate toxicity within 36 months of intervention start: two infusions of the monoclonal anti-CD20 antibody Rituximab (Mabthera®) (500 mg/m^2, max 1000 mg per infusion) given at two weeks' interval, followed by maintenance infusions of Rituximab (500 mg/m^2, max 1000 mg per infusion) at 3, 6, 10 and 15 months.

- functional MRI of the brain after standardized cognitive stress, before intervention and after 12 months follow-up, in 10 of the included patients; please refer to separate protocol section.

<u>**AMENDMENT dated 08.12.11:**</u> *For patients who have taken part in the KTS-2-2010 or KTS-3-2010 studies and experienced a slow and gradual improvement, but who could not be classed as clinically major responders according to the predefined criteria after 12 months follow-up (including five Rituximab infusions), we wish to intensify the Rituximab treatment during the following year. The aim is to investigate whether these patients can still achieve a clinically major response. We wish to administer up to 6 additional Rituximab infusions (500 mg/m^2, max 1000 mg) with at least two months intervals, during the following 12 months.*

DESIGN
Open phase II study. Single center.

INCLUSION OF PATIENTS
We will include up to 30 evaluable patients with Chronic Fatigue Syndrome (CFS). According to the research protocol for the completed clinical study KTS-1-2008, all 15 patients who were included in KTS-1-2008 and received the placebo intervention will be offered inclusion in this study, if they still meet the inclusion criteria and there are no contraindications to a rituximab intervention.

Patients who have received rituximab treatment in KTS-1-2008 will be offered inclusion no sooner than 12 months after the first treatment. This group will include patients who have experienced a clinical response followed by symptom relapse, and who have been clinically stable during the last 3 months before inclusion in the new study.
We also aim to include 2-3 participants from KTS-1-2008 where we believe, based on clinical assessment and patient reports, that the rituximab treatment had some effect on the symptoms, but no clinical response was recorded (late and transient improvement which does not meet the response criteria). Our hypothesis is that these patients may benefit from prolonged B-cell depletion (dose-response effect).
Additionally, we will treat two patients from KTS-1-2008 who received rituximab treatment but experienced no clinically relevant response, based on the same assumption that the lack of response may be related in some patients to an insufficient dose and duration of B-cell depletion.
We will also include additional patients who have been diagnosed with CFS following a neurological assessment, and who meet the study inclusion criteria.

INCLUSION CRITERIA
-Patients with CFS according to the Fukuda 1994 criteria 1994 [6], diagnosed by a neurologist.
-Age 18 to 66 years.
-Signed informed consent.

EXCLUSION CRITERIA
-Patients suffering from fatigue who do not meet the diagnostic criteria for CFS, who have symptom duration of less than 6 months or where the assessment uncovers other pathology which may cause the patient's symptoms.
-Pregnancy or lactation. Positive pregnancy test (urine).
-Previous malignant disease (except basal cell carcinoma in skin or cervical dysplasia).
- previous severe immune system disease, except autoimmune diseases such as e.g. thyroiditis or diabetes type I.
- previous long-term systemic immunosuppressive treatment (such as azathioprine, cyclosporine, mycophenolate mofetil, except steroid courses for e.g. obstructive lung disease).
-Severe endogenous (primary) depression.
-Lack of ability to adhere to protocol.
-Known multi-allergy with clinical risk from rituximab infusions.
-Reduced kidney function (serum creatinine > 1.5x upper normal value).
-Reduced liver function (serum bilirubin or liver transaminases > 1.5x upper normal).
-Known HIV infection, or evidence of ongoing active and relevant infection.

BACKGROUND FOR PROJECT AMENDMENT dated 08.12.2011
The main purpose of the KTS-2-2010 and KTS-3-2010 studies was to investigate whether maintenance treatment with Rituximab would result in prolonged symptom relief compared to two infusions given at two weeks' interval, as in the KTS-1-2008 study. Thus, the main study focus is on maintenance and dose-response relationships

aiming to optimize the medical intervention for Chronic Fatigue Syndrome/Myalgic Encephalopathy (CFS/ME). The results from the KTS-1-2008 study have now been published in Plos One (Fluge et al, Plos One, 2011). The study was double-blind, randomized and placebo controlled, and two groups of 15 patients received Rituximab 500 mg/m^2 (max 1000 mg), two infusion two weeks apart, or an equivalent infusion of saline, with follow-up for 12 months. Our main finding was a significant time by intervention group interaction for self-reported symptom scores in favor of the Rituximab group. The differences between the groups were most evident between 6-10 months after intervention start date (i.e. secondary endpoints), and the primary endpoint at 3 months was negative. The Overall Response Rate showed that 10 out of 15 patients in the Rituximab group and 2 out of 15 in the placebo group experienced a clinically significant improvement during follow-up.
In other words, one third of the Rituximab group had no significant response to B-cell depletion with Rituximab (two infusions two weeks apart). For some non-responders, the dose of Rituximab may have been insufficient, and in the ongoing study (KTS-2-2010) with Rituximab, two infusions two weeks apart, followed by maintenance infusions after 3, 6, 10 and 15 months (26 patients) we included two patients who took part in the first study but achieved no response. One of these patients now shows evidence of major response on all CFS/ME symptoms during maintenance treatment, which indicates that the dose-response relationship is relevant to some patients.

26 patients have been included in the current KTS-2-2010 maintenance study. All patients have now been through at least 10 months follow-up. The preliminary data indicate that our hypothesis on longer lasting response after maintenance treatment was correct. For some patients, however, there is still no sign of response after 12 months follow-up. These are characterized as non-responders, and we are now working on a protocol application to the Regional Ethical Committee regarding the experimental treatment of some of these patients by alternative strategy (see separate application).
There is also one group (approx. 5 out of 26 patients included in KTS-2-2010) who have not achieved the defined response criteria for effect on CFS/ME symptoms, but where both the patients and the investigators agree that B-cell depletion using Rituximab has nevertheless had a beneficial effect on the symptoms. This group includes patients with very long history of ME/CFS (> 10 years). If our hypothesis that CFS/ME may be a form of autoimmune condition is correct, some patients may have very high levels of putative autoantibodies initially, so that the patients fail to eliminate these (half-life 3-5 weeks) despite long lasting (12 months) B-cell depletion. In other patients, it may be that presumed autoantibodies are produced by fully matured plasma cells which are affected to a smaller extent by anti-CD20 antibodies, while such treatment is more efficient where autoantibodies are produced by less mature plasmablasts. This mechanism for non-response to Rituximab has been demonstrated in other autoimmune conditions.

AMENDMENT dated 08.12.11: *For patients who have taken part in the KTS-2-2010 or KTS-3-2010 studies and experienced a slow and gradual improvement, but who could not be classed as clinically significant responders according to the predefined criteria after 12 months follow-up (including five Rituximab infusions), we wish to intensify the Rituximab treatment during the following year. The aim is to investigate whether these patients can still achieve a clinically major response. We*

wish to administer up to 6 additional Rituximab infusions (500 mg/m^2, max 1000 mg) with at least two months intervals, during the following 12 months.

DATA MANAGEMENT AND STATISTICAL ANALYSIS

The demographic and clinical characteristics of the patient groups will be described. We will focus on longitudinal changes in health-related quality of life. We will record changes for each individual patient and for the patient cohort, with emphasis on changes over time. Statically and clinically significant response will be assessed. The patients will complete a self-report form for symptom change every two weeks throughout the follow up period.

The SF-36 (Short Form 36) questionnaire will also be used for the purpose of patient self-reporting. The questionnaire will be completed by patients before intervention and every 4 months during follow-up.
Doctor's assessment of symptoms and toxicity will be performed before treatment and 3, 6, 10, 15, 20, 24, 32 and 36 months after intervention start date, and will be recorded on a separate form.

Due to the lack of diagnostic laboratory tests or other specific tests for this illness, adequate registration will depend on patient symptom variables. Thus the effect variables will relate to the four main symptom categories in CFS: Fatigue, Pain, Cognitive symptoms and Other symptoms (including autonomic symptoms). The patients will also record how they experience the illness as a whole, and the perceived impact of the intervention on their quality of life (refer to patient self-report form). Each patient will also fill inn a registration form (before intervention) scoring his/her present symptoms. Only symptoms which affect the individual patient at baseline according to the registration form (within a main category: Fatigue, Pain, Cognitive, Other) will be analyzed for changes at endpoints. Any symptom change, or lack of such, compared to baseline is recorded by the patients every two weeks during follow-up. A score for each of the main categories is calculated, based on the mean scores for each category of symptom on the form.
Longitudinal changes in specific symptoms (within each main category) over time will be analyzed and compared to baseline values for each patient and for the entire cohort.
After data analysis at 14 months, 24 months and 36 months we will consider the publication of interim data. We will analyze data for the entire cohort, but also perform separate analyses not including data for the patients with no clinical response in the KTS-1-2008 study, who are nevertheless included in this study (please refer to "Inclusion of patients" above; these patients were included in order to investigate whether treatment dose/maintenance could be relevant to the lack of response in KTS-1-2008).
The patients randomized to the placebo arm of KTS-1-2008 have completed a 12 months registration period without receiving any active intervention, and any symptom change after Rituximab infusions will be comparable to the follow-up data after the placebo intervention. Patients who received Rituximab during KTS-1-2008, experiencing response and subsequent relapse, can be compared to their own reports after the KTS-1-2008 intervention (i.e. Rituximab treatment with or without maintenance infusions). We will also record the extent to which the patients are able

to participate in educational activities, employment or social life during the study period (36 months).

Excel computer software will be used for data input, and SPSS for the purpose of statistical analysis. Data input from questionnaires to Excel will be performed by Øystein Fluge, and monitored/double-checked by Olav Mella. Data monitoring will be performed as and when required.

Forms with original data will be stored at the trial sites for 15 years after final report is issued. All computer files will be stored accordingly at the Dept. of Oncology. At each clinical visit, the doctor will dictate an entry in the hospital's electronic medical journal.

INFORMATION VISIT AT THE ONCOLOGY DEPT. OUTPATIENT CLINIC

The patients will attend a consultation at the Dept of Oncology, outpatient clinic for assessment, information and written consent.

EXAMINATION AND REGISTRATION AFTER SIGNED INFORMED CONSENT, BEFORE INCLUSION AND INTERVENTION

Clinical assessment: Assessment of CFS symptomatology and registration of symptom severity on the relevant form. Exclusion of other medical conditions which may cause considerable fatigue such as: hypothyreosis, adrenal insufficiency, malignancy, chronic infections, lung disease, angina pectoris, heart failure, kidney failure, liver disease, other neurological diseases (multiple sclerosis, brain tumors, cerebrovascular disease), endogenous depression or other psychiatric conditions associated with fatigue.

Laboratory tests:

-Hb, ESR, WBC differential, platelet count, MCV.
-Ferritin, s-Fe, Vitamin B12, Folate, Na, K, Ca, Mg, Phosphate, Glucose.
-Creatinine, Carbamide, Urate, Triglycerides, Total Cholesterol, HDL cholesterol.
-ALAT, ALP, GGT, Bilirubin.
-CRP, Albumin, Total Protein, INR.
-Pregnancy test (HCG) for women of childbearing age.

Immunology:

-Serum Protein Electrophoresis, Quantitative Immunoglobulins with IgG, IgG subclasses, IgM, IgA.
-Immunophenotyping of lymphocytes in peripheral blood.
-transglutaminase (Celiac Disease Test), Antinuclear Antibody Test, Rheumatoid Factor, Thyroid Antibodies, Adrenal Antibodies, Cardiolipin antibodies.

Endocrinology:

-fT4, TSH, Prolactin, Cortisol/ACTH.

Microbiology:

-Serology for EBV, CMV, HSV, VZV, Enterovirus, Parvovirus B19, Adenovirus.

Patients who took part in the KTS-1-2008 study and who completed all tests before inclusion in KTS-1-2008, will not be subject to repeated antibody and endocrinology tests unless there is clinical suspicion of pathological changes.

Biobank blood tests (see separate section).

ADMISSION TO THE ONCOLOGY WARD FOR RITUXIMAB INFUSION
The patients will have completed clinical assessment, inclusion and all relevant tests before being admitted for treatment.
The patients will be admitted to the Dept. of Oncology on the morning of treatment. Patients will receive Rituximab 500 mg/m^2, max. dose 1000 mg, diluted in NaCl 0.9 % to a concentration of 2 mg/ml. The second infusion, at 10-14 days after the first, and maintenance infusions after 3, 6, 10 and 15 months, will all contain the same dose of Rituximab as the first infusion.
On the day of treatment all patients will receive the following pre-medication: Cetirizine 10 mg x 1 po, Paracetamol 1 g x 2 po, and Dexamethason 8 mg x 1 po.
The intravenous infusion speed will follow the guidelines for Rituximab infusions in lymphoma treatment. A nurse shall be present and monitor blood pressure, heart rate and saturation as specified (see appendix). If the first infusion is administered without any symptoms, the infusions will be given more rapidly, according to guidelines for Rituximab infusions in lymphoma patients (see appendix).
If the patient has no sign of clinical response according to medical assessment before the 10 month infusion, the planned maintenance infusions at 10 and 15 months will be cancelled. Patients will continue follow-up at the Dept. of Oncology,outpatient clinic as detailed in the protocol with regards to symptom change and toxicity.

4 weeks after treatment:
Laboratory tests:
Hb, WBC differential, platelet count, ALAT, ALP, GGT, Bilirubin, Creatinine, CRP.

EXAMINATION AND REGISTRATION AT 3, 6, 10, 15, 20, 24, 32 AND 36 MONTHS FOLLOW-UP

Clinical assessment with registration. Patients hand in completed self-report forms.
Laboratory tests:
-Hb, ESR, WBC differential, platelet count, Na, K, Ca, Phosphate, Glucose, Creatinine, Urea, Urate, ALAT, ALP, GGT, LD, Bilirubin, CRP, Albumin, Total Protein.
Immunology:
-Serum Protein Electrophoresis, Quantitative Immunoglobulins with IgG, IgG subclasses, IgM, IgA
-Immunophenotyping of lymphocytes in peripheral blood.
Biobank blood tests (see separate section).

At each visit the doctor will perform a clinical assessment and dictate an entry in the patient's electronic records. The patient's completed self-report form (recorded every two weeks) and SF-36 questionnaire for the relevant time period are copied and stored in the patient's case file.
Patients who have access to the internet may send scanned anonymized copies the complete self-report forms by e-mail to the study management every 1-2 months, in order to facilitate data registration and input.

SELF-REPORT FORMS FROM PATIENTS

-Each patient will fill in a self-report form for CFS symptoms before treatment (week 0) and thereafter every two weeks for 36 months. Copies of the form are collected at follow-up visits at 0, 3, 6, 10, 15, 20, 24, 30, and 36 months.
The same self-report form has been used in the KTS-1-2008 study, and both doctors and patients expressed that the form recorded longitudinal symptom changes adequately. The SF-36 quality of life questionnaire is used for self-reporting of the patients' symptoms. This form shall be completed every 4 months until 36 months after intervention. SF-36 is a generic form, which has been used frequently during the last 15 years. [7, 8]. A Norwegian, validated version is available [9]. The use of any medications will be recorded. Any side effects, including the occurrence of infections, are recorded.

TOXICITY

Any unexpected serious adverse event which is deadly or life-threatening will be reported to the Regional Ethical Committee (REC) and the Norwegian Medicines Agency (NOMA) within 7 days of the study management being notified of the event. Other unexpected serious adverse events will be reported within 15 days. Any remaining adverse events will be reported in a combined final report. Only SUSARs will be reported as an individual report. The study management – Head of Dept. Prof Olav Mella and Øystein Fluge, MD, are responsible for reporting SAEs to the REC and NOMA. The safety aspect of the study will be supervised by Prof. Olav Dahl at the Dept. of Oncology, HUS. He will take no part in the clinical evaluation of patients.
Work-up before inclusion should exclude patients with suspected immunodeficiency disorders or ongoing active and relevant viral infections. However, the cause of ME/CFS is as yet unknown, and we cannot exclude the possibility that some patients may suffer an ongoing active viral infection, which is undetectable at the work-up, and where B-cell depletion could theoretically cause a clinical deterioration.
We have not observed any patients in the pilot series or in the KTS-1-2008 study (in total 33 patients, 18 treated with Rituximab) whom, during up to 2 years' follow-up, have reported a decline in their CFS condition after intervention. Patients will receive information on the safety aspects orally and in the letter of information.

PATIENT WITHDRAWAL DURING STUDY

The patients will be informed verbally and in the written patient information that they may withdraw from the study at any time, without having to state the reason for their decision. The medical reasons for withdrawing a patient from the study may be serious events such as severe allergic reactions during or short time after the infusion. Patients who withdraw from the study due to intercurrent or other diseases or any other reasons, will be followed by their general practitioner according to usual CFS guidelines. If possible, we will attempt to obtain toxicity data from patients who have withdrawn during the study.

BIOLOGICAL SPIN-OFF STUDIES

Our studies show that B-lymphocyte depletion using an anti-CD20 monoclonal antibody (rituximab) may have a therapeutic effect in CFS. We will systematically

extend the existing biobank with blood samples from the patients at baseline and throughout 36 months follow-up. The biobank will be a starting point for further research into the pathogenesis of ME/CFS. The mechanisms behind the disease must be charted, and a specific and sensitive biomarker is greatly needed.

In addition to laboratory tests as specified above, blood samples for biobank and research will be collected (following written informed consent) before treatment and at follow-up after 3, 6, 10, 15, 20, 24, 30 and 36 months. We will collect serum, plasma and whole blood at specified time points, as well as blood samples on special tubes for DNA and RNA preservation and protein from lymphocyte fractions. Following written informed consent, some patients may be requested to donate samples of cerebrospinal fluid (frozen after collection) before intervention and possibly after 12 and 24 months follow-up.
Biological spin-off studies will be supervised by Olav Mella, Øystein Fluge and Prof. Olav Dahl (Dept. of Oncology at HUS and Section of Oncology, Institute of Medicine at the University of Bergen) in collaboration with research scientist Ove Bruland, Ph.D. (Centre for Medical Genetics and Molecular Medicine at HUS) and Kristin Risa, M.Sc. (Dept. of Oncology). Researchers from the Departments of Oncology, Microbiology and Immunology, and Neurology will participate in substudies according to mutual agreement.

FUNCTIONAL MRI OF THE BRAIN

In collaboration with the senior consultant Gesche Neckelmann (Dept. of Radiology, HUS) and Prof. Kenneth Hugdahl (Institute for Biological and Medical Psychology, UiB) we wish to conduct a substudy using a functional MRI and MR spectroscopy of the brain. The fMRI exam involves a standardized cognitive stress test focusing on memory, and will be performed before intervention and after 12 months follow-up. Ten patients included in either KTS-2-2010 or KTS-3-2011 will be asked to participate in this study. The MRI of the brain does not involve injection of contrast fluid or any other invasive procedure.

FUNDING

The study is investigator initiated and is funded through the research budget at the Dept. of Oncology, HUS. There is no external sponsor for the clinical study. Research originating from the biobank (”Pathogenesis and biomarkers in Chronic Fatigue Syndrome) receives financial support from Western Norway Regional Health Authority for the period 2010-2012.

PUBLICATION

The results – positive or negative – from the clinical study will be published in a reputable medical journal. Co-authorship and order of authors will comply with the Vancouver guidelines.

APPLICATIONS FOR APPROVAL

Applications for approval will be sent to:
-The Regional Ethical Committee.

-The Biobank Register (extension of existing biobank).
-EudraCT.
-The Norwegian Medicines Agency.

REFERENCES (refer to the project description for a complete list of references)

1. Fluge O, Mella O: **Clinical impact of B-cell depletion with the anti-CD20 antibody rituximab in chronic fatigue syndrome: a preliminary case series**. *BMC Neurol* 2009, **9**(1):28.
2. Lombardi VC, Ruscetti FW, Das Gupta J, Pfost MA, Hagen KS, Peterson DL, Ruscetti SK, Bagni RK, Petrow-Sadowski C, Gold B, Dean M, Silverman RH, Mikovits JA: **Detection of an Infectious Retrovirus, XMRV, in Blood Cells of Patients with Chronic Fatigue Syndrome**. *Science* 2009.
3. Groom HC, Boucherit VC, Makinson K, Randal E, Baptista S, Hagan S, Gow JW, Mattes FM, Breuer J, Kerr JR, Stoye JP, Bishop KN: **Absence of xenotropic murine leukaemia virus-related virus in UK patients with chronic fatigue syndrome**. *Retrovirology* 2010, **7**(1):10.
4. van Kuppeveld FJ, Jong AS, Lanke KH, Verhaegh GW, Melchers WJ, Swanink CM, Bleijenberg G, Netea MG, Galama JM, van der Meer JW: **Prevalence of xenotropic murine leukaemia virus-related virus in patients with chronic fatigue syndrome in the Netherlands: retrospective analysis of samples from an established cohort**. *Bmj* 2010, **340**:c1018.
5. Erlwein O, Kaye S, McClure MO, Weber J, Wills G, Collier D, Wessely S, Cleare A: **Failure to detect the novel retrovirus XMRV in chronic fatigue syndrome**. *PLoS One* 2010, **5**(1):e8519.
6. Fukuda K, Straus SE, Hickie I, Sharpe MC, Dobbins JG, Komaroff A: **The chronic fatigue syndrome: a comprehensive approach to its definition and study. International Chronic Fatigue Syndrome Study Group**. *Ann Intern Med* 1994, **121**(12):953-959.
7. Ware JE, Jr., Sherbourne CD: **The MOS 36-item short-form health survey (SF-36). I. Conceptual framework and item selection**. *Med Care* 1992, **30**(6):473-483.
8. Myers C, Wilks D: **Comparison of Euroqol EQ-5D and SF-36 in patients with chronic fatigue syndrome**. *Qual Life Res* 1999, **8**(1-2):9-16.
9. Loge JH, Kaasa S, Hjermstad MJ, Kvien TK: **Translation and performance of the Norwegian SF-36 Health Survey in patients with rheumatoid arthritis. I. Data quality, scaling assumptions, reliability, and construct validity**. *J Clin Epidemiol* 1998, **51**(11):1069-1076.
