# Supplementary figures and images for: B-Lymphocyte Depletion in Myalgic Encephalopathy/ Chronic Fatigue Syndrome. An Open-Label Phase II Study with Rituximab Maintenance Treatment
Source: PLoS One. 2015 Jul 1;10(7):e0129898. doi: 10.1371/journal.pone.0129898 (PMC4488509; doi:10.1371/journal.pone.0129898)

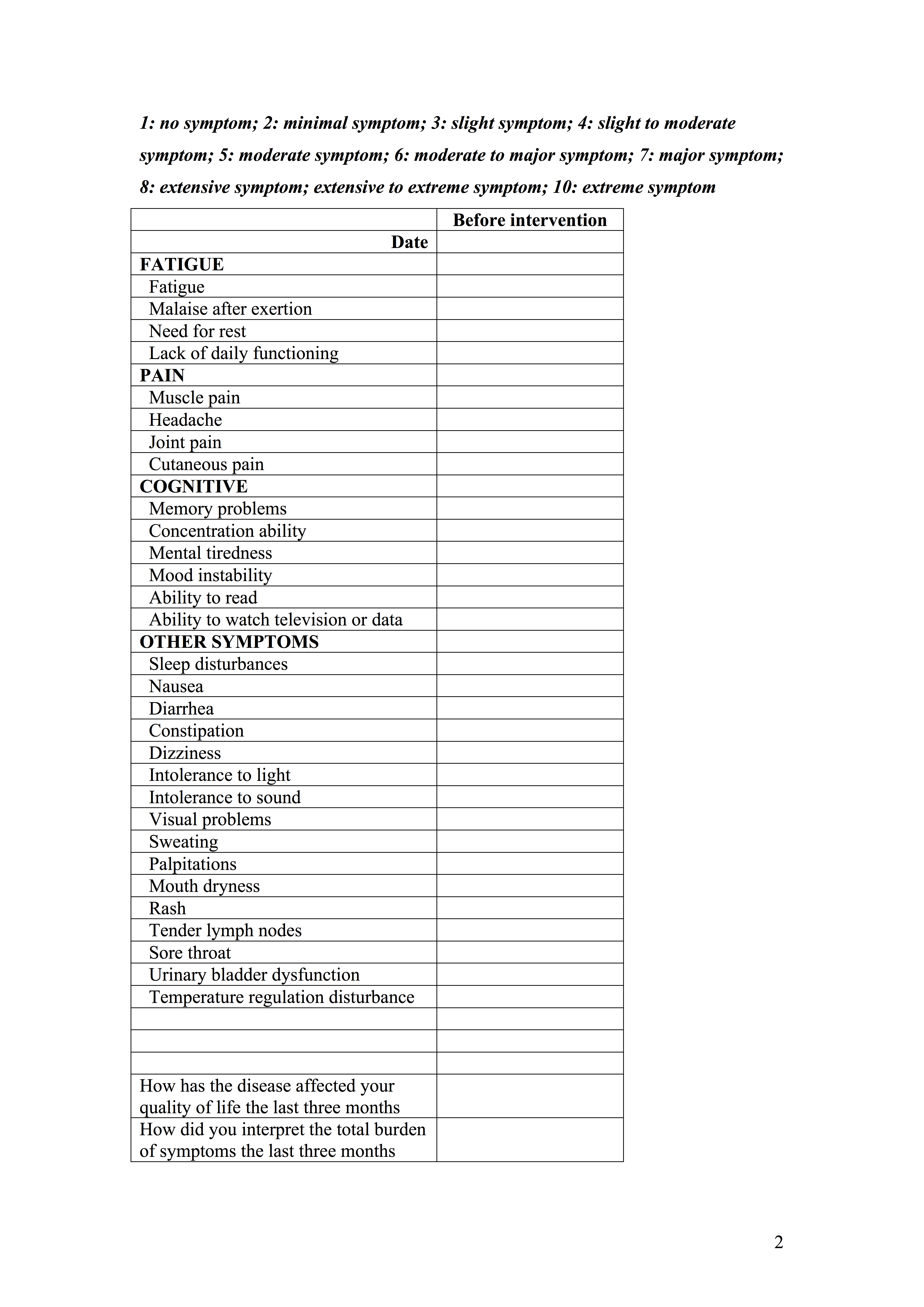

Supplement: S1 Fig — Before intervention, the patients assessed their ME/CFS disease and recorded their symptoms during the preceding three months period, using the scale 1–10 (1: no symptom; 5: moderate symptom; 10: very severe symptom). (TIF) [file pone.0129898.s003.tif]

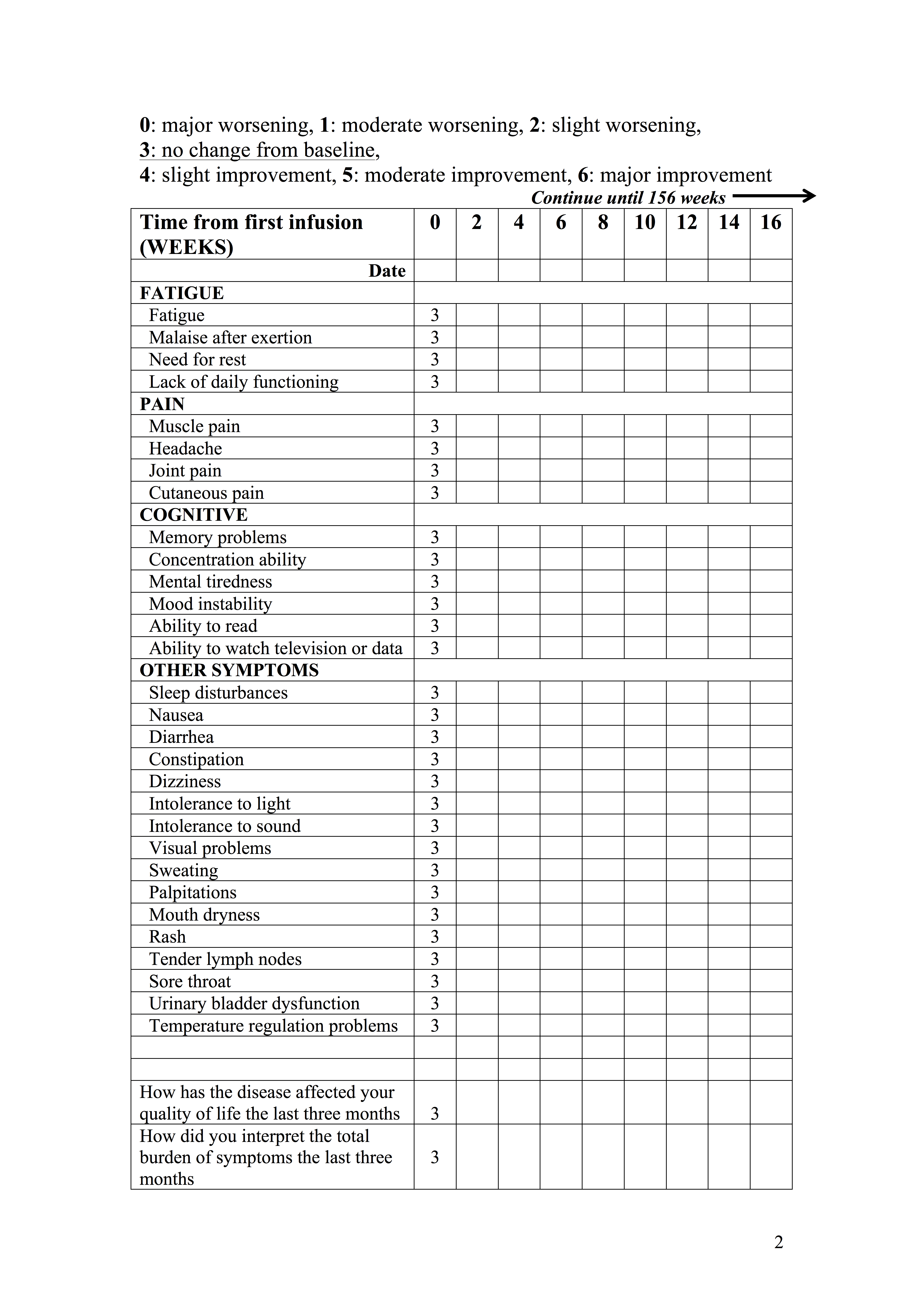

Supplement: S2 Fig — During 36 months follow-up, the patients recorded symptom changes every two weeks, always as compared to baseline. The scale for the follow-up form was 0–6 (0: Major worsening; 1: Moderate worsening; 2: Slight worsening; 3: No change from baseline; 4: Slight improvement; 5: Moderate improvement; 6: Major improvement). (TIF) [file pone.0129898.s004.tif]

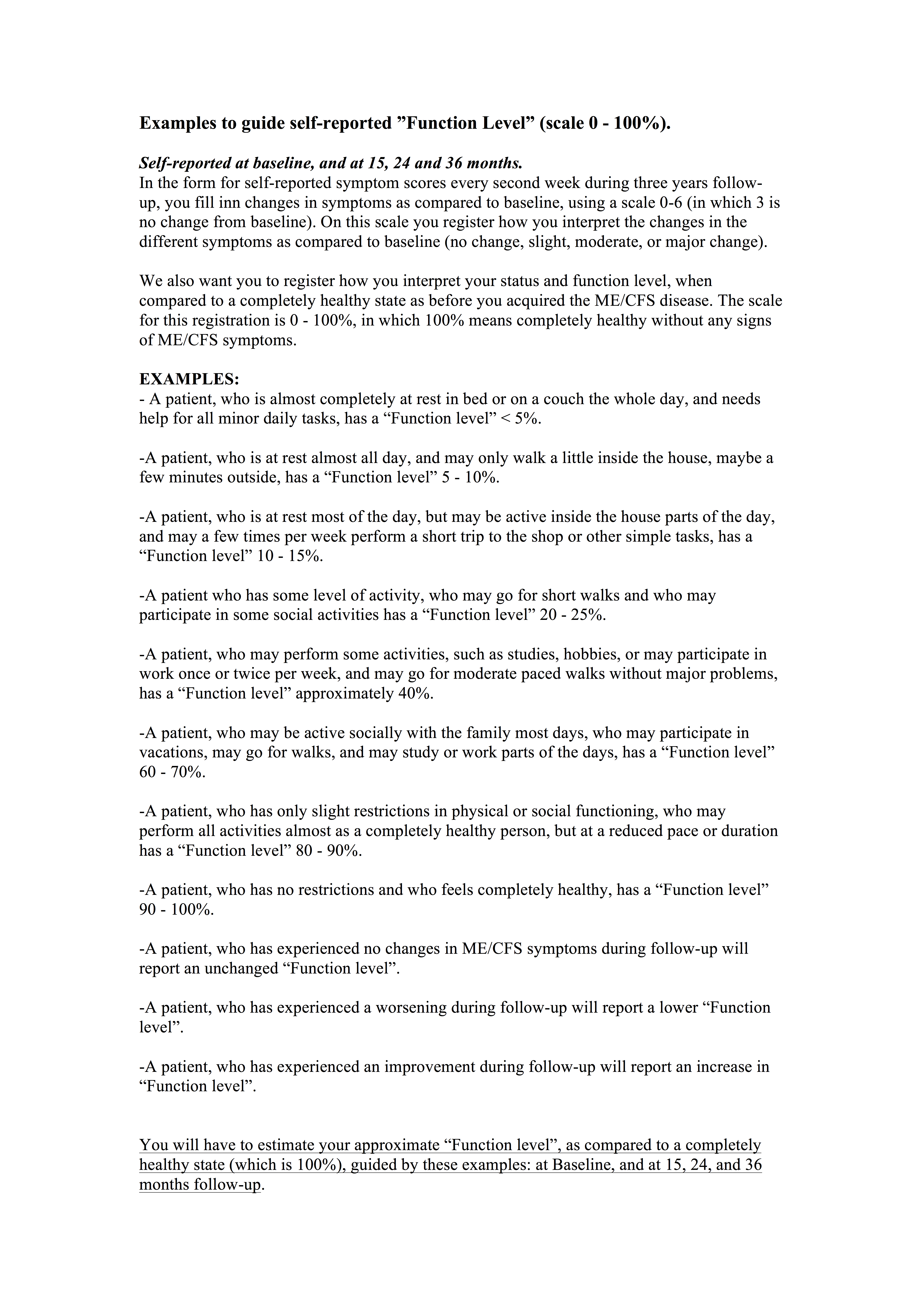

Supplement: S3 Fig — The patients assessed their Function level (scale 0–100%) in which 100% denoted completely healthy as before the patient acquired ME/CFS, according to examples in this form. The Function levels were assessed at baseline, and at 15, 24 and 36 months follow-up. (TIF) [file pone.0129898.s005.tif]

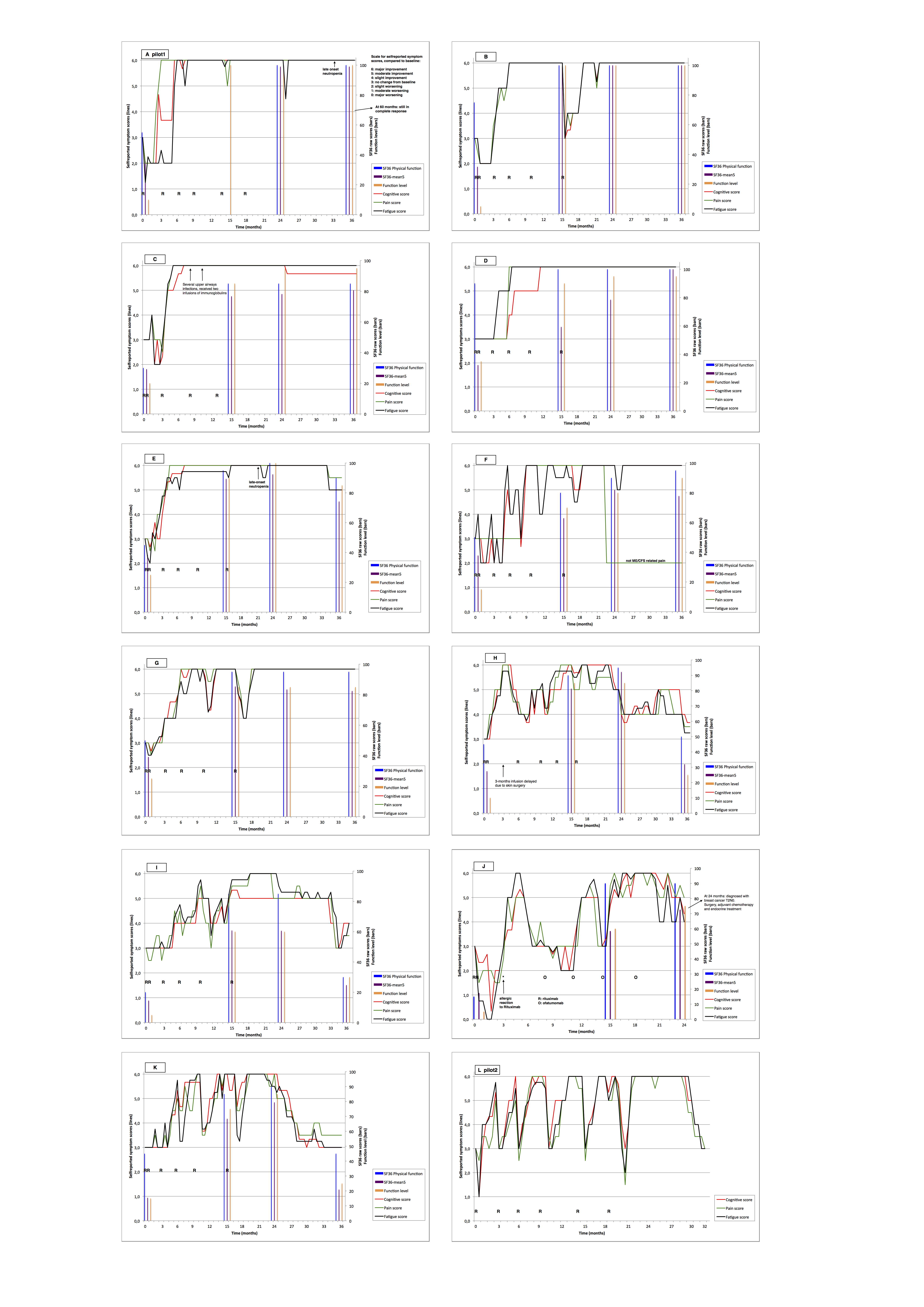

Supplement: S4 Fig — Panels A-L show follow-up data for each of 12 patients with major clinical responses during 36 months follow-up. In each panel the lines represent self-reported symptom scores. Every second week, the patients recorded symptom changes, always compared to baseline, in a separate form (S2 Fig). Fatigue score (black line) was calculated every second week as the mean of four fatigue-related symptoms (Fatigue, Post-exertional malaise, Need for rest, Daily functioning). Cognitive score (red line) was calculated as the mean of three symptoms (Concentration ability, Memory disturbance, Mental tiredness). Pain score (green line) was calculated as the mean of the two dominant pain symptoms (if pre-treatment level ≥ 4). The vertical bars (scales 0–100) represent the SF-36 raw scores for Physical function (blue bars), “SF-36mean5” (i.e. mean of raw scores for Physical function, Bodily pain, Vitality, General health, Social function) (purple bars), and self-reported Function level according to a form with examples (S3 Fig) (orange bars), at baseline, and at 15, 24 and 36 months follow-up. “R” in the panels indicates time points for rituximab infusions. (TIF) [file pone.0129898.s006.tif]

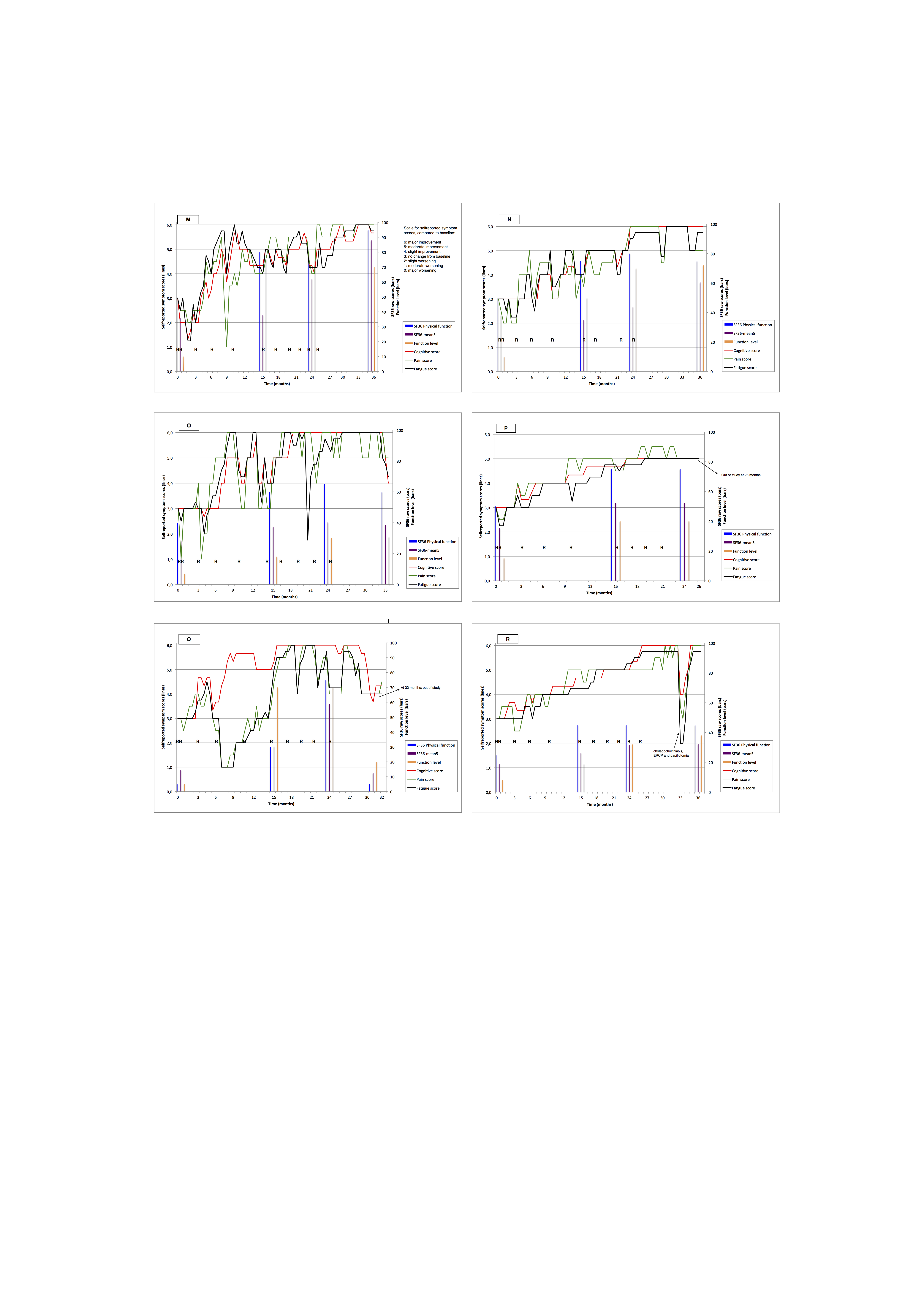

Supplement: S5 Fig — Panels M-R show follow-up data for each of for six patients receiving additional rituximab-infusions, according to a study amendment, including two patients with major response, and four patients with moderate response. See legend to S4 Fig. (TIF) [file pone.0129898.s007.tif]

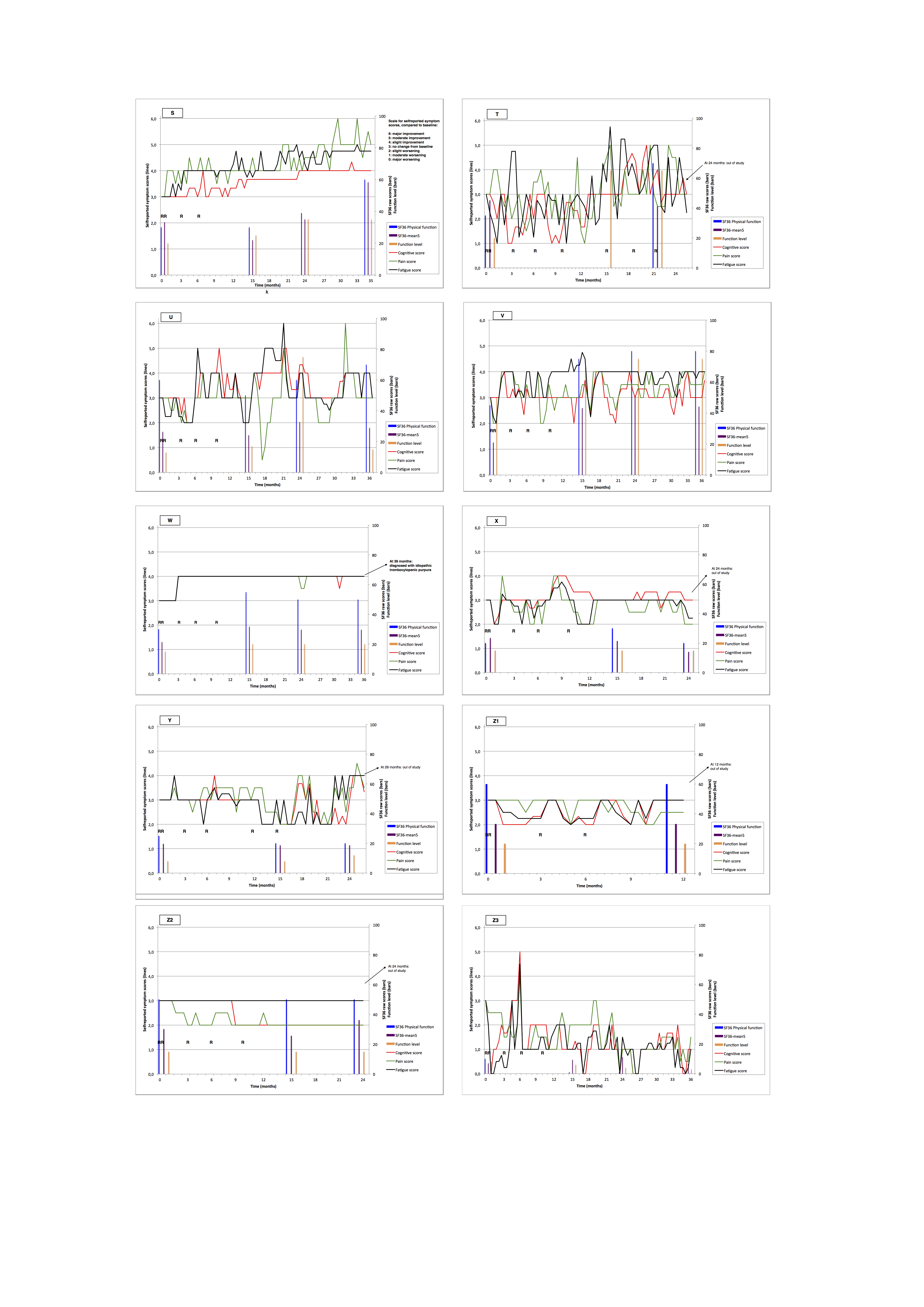

Supplement: S6 Fig — Panels S-Z3 show follow-up data for each of 10 patients with no clinically significant response, including one patient with “marginal” response, and nine patients with no response during follow-up. See legend to S4 Fig. (TIF) [file pone.0129898.s008.tif]

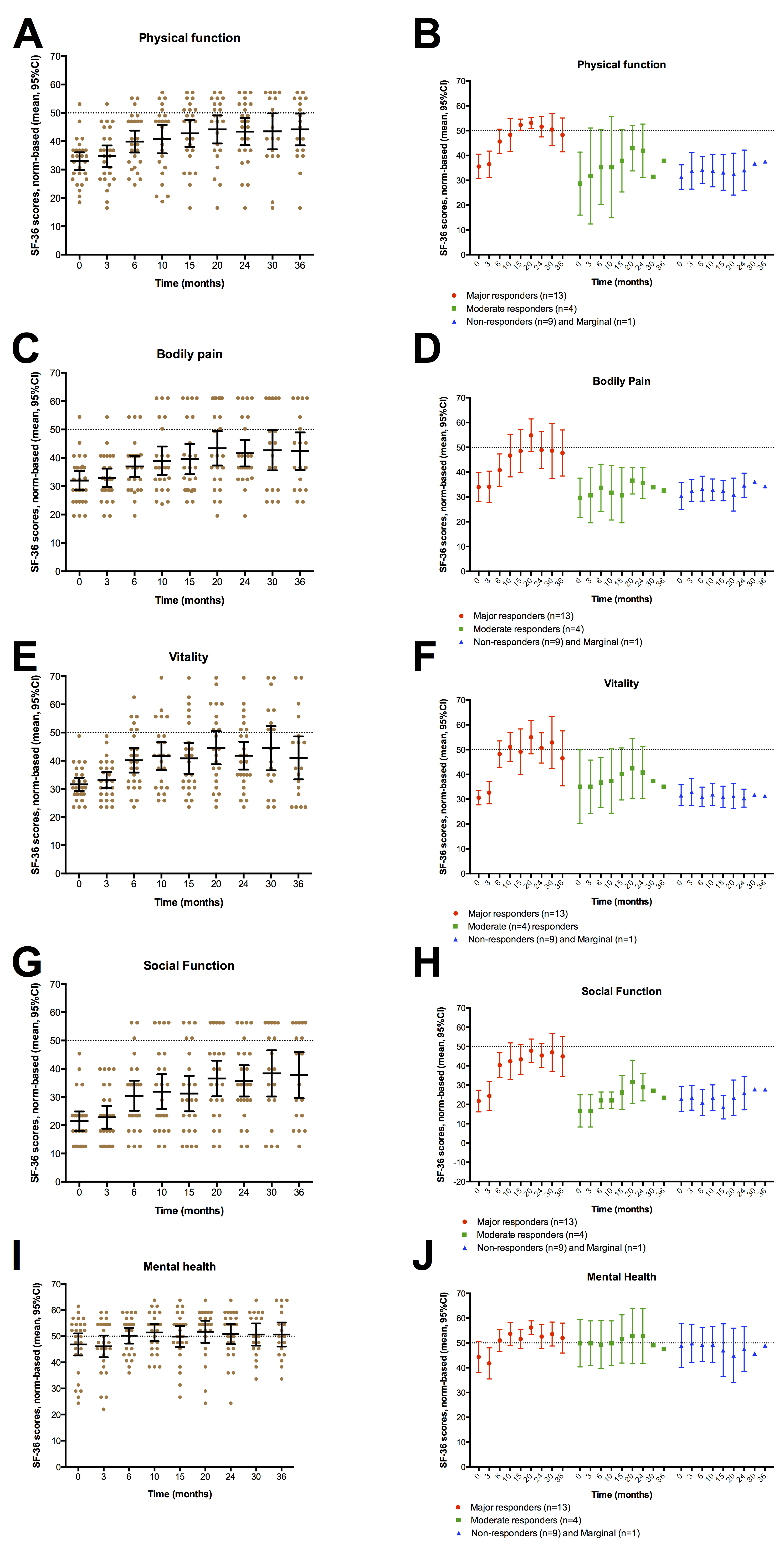

Supplement: S7 Fig — SF-36 (Norwegian ver. 1.2) forms were recorded at baseline and at 3, 6, 10, 15, 20, 24, 30 and 36 months. Norm-based SF-36 scores (according to US 1998) are shown for 27 patients included in the KTS-2-2010 study, for the subdimensions Physical function (panel A), Bodily pain (panel C), Vitality (panel E), Social function (panel G) and Mental health (panel I). In panels B, D, F, H and J the corresponding SF-36 norm-based scores are shown separately for 13 major responders, four moderate responders, and 10 patients with no clinical significant response (one marginal responders and nine non-responders). The horizontal line in each panel denotes the approximate population means for SF-36 norm-based scores (50). One pilot patient (major responder, withdrew after 32 months) did not fill in SF-36 forms. One included patient did not receive induction rituximab infusions due to an allergic reaction to the first infusion, and did not fill in SF-36 forms. One major responder was diagnosed with a T2N0 breast cancer after 24 months follow-up and withdrew from study to start cancer treatment. Out of four moderate responders, one withdrew from the study after 25 months, and one after 32 months. Out of 10 patients with no clinically significant response one withdrew from study after 12 months, and four patients after 24–26 months follow-up. (TIF) [file pone.0129898.s009.tif]
